# Biochemical and Physiological Effects of Galanin in Health and Disease

**DOI:** 10.1096/fj.202504477R

**Published:** 2026-01-17

**Authors:** Patrick Mireles, Yubo Wang, Kathryn Rhodes, Sharon DeMorrow

**Affiliations:** ^1^ Division of Pharmacology and Toxicology College of Pharmacy, University of Texas at Austin Austin Texas USA; ^2^ Department of Internal Medicine Dell College of Medicine, University of Texas at Austin Austin Texas USA

**Keywords:** galanin, galanin receptors, ligands, neurotransmitter agents, peptide hormones, signal transduction

## Abstract

Galanin is a biologically active peptide discovered in 1983 from the intestines of pigs. Discovered by Doctors Tatemoto, Rökaeus, Jörnvall, McDonald, and Mutt, it was found to contract smooth muscle tissue in rat intestine and produce hyperglycemia in dogs. Since its discovery, research into galanin has revealed a wide array of effects in numerous organ systems. As these effects have been uncovered, there has been growing interest in the galanin system as a therapeutic target. Targeting galanin has proven difficult as it influences much of the body, leading to challenges in identifying the source of observed changes and, moreover, selecting those sources as targets. A critical tool in overcoming these challenges is a cohesive understanding of galanin's broad effects in various organ systems. Galanin and galanin receptor expression, receptor and ligand affinity, biochemical signaling paths, and physiological effects of galanin remain under investigation. As research into this field continues, greater appreciation of the complexity of galanergic signaling is critical to elucidate galanin's role in health. This review seeks to provide insight into these aspects and provide researchers with the knowledge needed to continue to expand investigations in the galanergic system.

## Graphical abstract

Galanin is a biologically active neuropeptide hormone that mediates its effects through three galanin receptors. Galanin signaling induces changes in glucose homeostasis amongst others and plays a dynamic role in the physiological health of many organ systems. Organ disruption, in turn, influences galanin signaling, potentially impacting other organ systems.

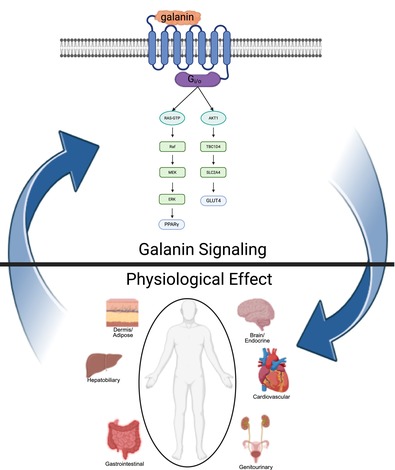

## 1 Introduction

Galanin is an endogenous neuropeptide discovered in 1983 [1] and has since been identified in most mammalian species. Galanin exerts its effects via three receptors, aptly named galanin receptors 1, 2, and 3 (abbreviated galR1, galR2, and galR3). Galanin and its receptors are expressed in most tissue types of the body, though they are especially present in the brain, gastrointestinal (GI), and reproductive organs. The galanin receptors are G‐protein‐coupled receptors (GPCRs), each of which binds specific g‐subunits. While some overlap exists, galanin signaling produces unique effects based on the tissue of expression and the individual galanin receptor subtype. Further, galanin has several sources, being excreted locally to exert autocrine functioning, at terminal synapses as a neurotransmitter, and excreted systemically as an endocrine hormone. Given the wide range of factors at play in galanin signaling, understanding these factors remains critical to confidently measure galanin's physiological effects.

## 2 Galanin—Structure and Function

**FIGURE 1:**
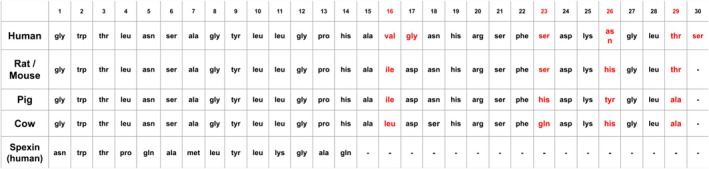
Galanin and spexin peptide amino acid sequences of various mammals. Thirty amino acid human sequences are shown first, followed by rat/mouse, pig, and cow 29 amino acid sequences. Human peptide C‐terminus terminates with a free acid; all others terminate with an amide. Differences in sequence are highlighted in red. Positions 16, 23, 26, and 29 possess the greatest heterogeneity. Positions 1–15 are completely conserved across the species shown. Spexin, shown at bottom, contains 14 amino acids and exhibits homogeneity with mammalian galanin at positions 2, 3, 9, 10, and 12. Spexin is cleaved at dibasic sites at both C‐terminus and N‐terminus.

Galanin is a 29–30 amino acid peptide that can be found in most mammalian species. Originally isolated from pigs, galanin has been well detailed in humans, rats, mice, and cows. Through sequencing, it has been found that galanin has a highly conserved sequence, with roughly 85% homology across species [2]. Figure 1 shows the peptide chain for various species and highlights the homology between species. It is useful to note that while most species have a 29 amino acid sequence terminating in an amide, humans possess a 30 amino acid sequence terminating in a free acid [2].

**FIGURE 2:**
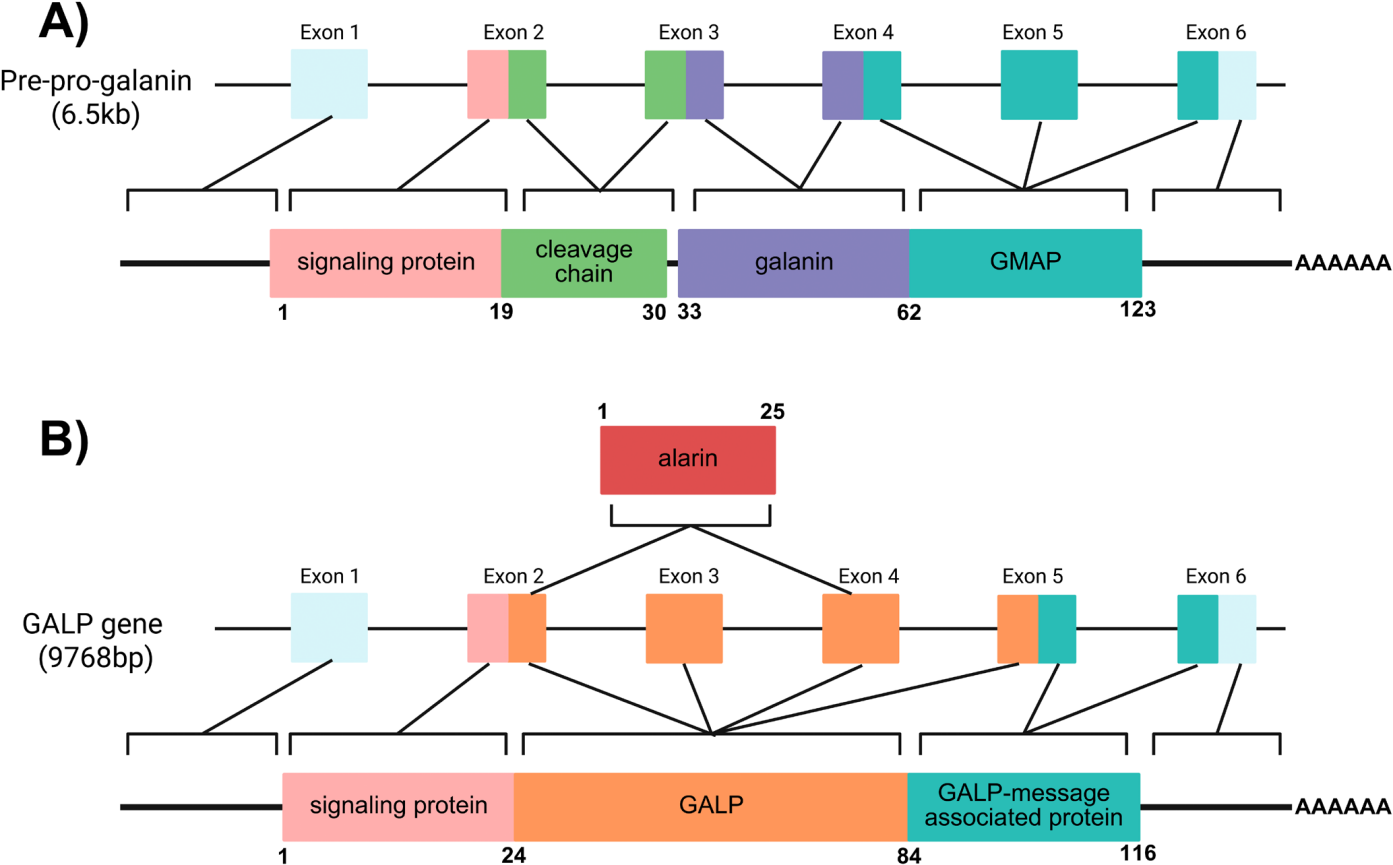
Depiction of pre‐pro‐galanin and GALP gene posttranscriptional and posttranslational splicing. (A) Pre‐pro‐galanin, a 6.5 kilobase pair gene containing six exons. Exon 1 encodes starting sequences, the signaling protein (19 amino acids) is encoded on the first portion of exon 2, and the cleavage chain (10 amino acids) is encoded and the second portion of exon 2 and the first portion of exon 3. Mature galanin (29 or 30 amino acids) is produced from the second portion of exon 3 and the first portion of exon 4. Mature GMAP (59 amino acids) is encoded on the second portion of exon 4, exon 5, and the first portion of exon 6. The remainder of exon 6 contains termination sequences. (B) GALP gene, a 9768 base pair gene containing six exons. Exon 1 encodes starting sequences. The first portion of exon 2 encodes the signaling protein (24 amino acids). Mature GALP (60 amino acids) is encoded by the second portion of exon 2, exons 3 and 4, and the first portion of exon 5. GALP‐message associated protein (32 amino acids) is encoded on the second portion of exon 5 and the first portion of exon 6, and the last portion of exon 6 encodes termination sequences. Alternative splicing combining the second portion of exon 2 and exon 4 yields alarin (25 amino acids), a unique biologically active peptide.

In humans, galanin is encoded by the *GAL* gene, a 6.5‐kilobase‐pair DNA sequence found on chromosome 11q13.3–q13.5 [3]. Preprogalanin, encoded by this sequence, is a 123 amino acid propeptide sequence. The *GAL* gene, as depicted in Figure 2a, contains six exons and five introns, of which parts of exons 2 and 3 encode galanin. Posttranslational cleavage of preprogalanin produces signaling protein, galanin, and galanin message associated protein (GMAP) [3]. The signaling protein represents the first 23 amino acids of preprogalanin, followed by a 10 amino acid‐long cleavage chain. Amino acids 33–62 of preprogalanin represent galanin, and amino acids 63 to the C terminus comprise GMAP.

*GAL* gene expressions vary greatly between organs. In humans, expression of *GAL* is greatest in the appendix, colon, brain, small intestine, and skin tissues [4]. Other gastrointestinal organs, the testes, prostate, bone marrow, and adipose tissue also express the *GAL* gene. In adult mice, *GAL* expression is greatest in adrenal, small intestine, large intestine, spleen, and stomach tissues [4]. Notable expression is also present in brain tissue, particularly in the frontal lobe, cerebellum, and thymus, as well as the lungs and other gastrointestinal organs.

Galanin is a multifunctional peptide, acting as a neurotransmitter, autocrine hormone, and endocrine hormone. Its peripheral neuronal activity appears to be predominately mediated via sympathetic innervations. Sympathetic stimulation results in increased galanin secretion from the liver while no change is observed in the gut [5]. In the pancreas, terminal synaptic regions secrete galanin to stimulate pancreatic islet cells. As an endocrine hormone, circulating galanin is proposed to originate from anterior pituitary secretion. This hypothesis comes from a transgenic mouse model in which the anterior pituitary gland overexpresses galanin which leads to increased plasma galanin levels [6], although this is not true for all species. The varied methodologies by which galanin stimulation can occur have led to challenges in identifying the exact pathway by which galanin acts.

The pharmacodynamics and pharmacokinetics of galanin are not well characterized across most species. There is, however, one report from 1992 that evaluated the characteristics of circulating galanin in pigs [7]. This report found that most circulating galanin was excreted from the intestinal tract and colon. In these pigs, it was also found that galanin possessed an average half‐life of 4.6 min in blood, an average distribution volume of 255.6 mL/kg, and found similar degradation results between plasma and blood [7]. Ethylenediaminetetraacetic acid alone and in combination with aprotinin preserved galanin stability in plasma and blood samples. In a quantitative analysis of galanin levels from gastrointestinal tissues, it was found that the transverse colon contained the most galanin compared to other sections [7]. The study also found that the kidney was a major point of galanin's removal from circulation. Galanin was not significantly detectable in urine samples, indicating renal clearance must be due to a mechanism other than filtration [7]. The study was performed using relative blood concentrations following first‐pass blood flow, and the authors noted that due to the complex circulation of the hepatic portal system they were unable to draw strong conclusions regarding liver excretion/absorption of galanin. Their findings highlight the importance of notating the formulation of galanin. The authors used porcine galanin in their porcine model and found that their results differed from observations from porcine galanin in human models. Much work is still needed to characterize the kinetics of galanin across other species.

## 3 Galanin Receptors

The galanin receptors are a set of three G‐protein‐coupled receptors with varied expression patterns depending on subtype. To date only the three galanin receptors have been identified and are respectively named galanin receptor type 1 (galR1), receptor type 2 (galR2), and receptor type 3 (galR3). Each receptor has its own associated gene, with the galR1 gene being encoded on chromosome 18q23 [4], galR2 encoded on 17q25.1 [4], and galR3 encoded on 22q13.1 [4]. GalR1 is predominantly expressed in adrenal and brain tissue, with notable expression in the prostate, thyroid, appendix, kidney, and adipose tissue [4]. GalR2 is expressed in nearly all tissues, with the greatest expression being in the bone marrow and appendix [4]. GalR3 is expressed mostly in the testes, though notable expression is found in the brain, pancreas, ovaries, prostate, bone marrow, and colon [4].

Activation of the galanin receptors is dependent on receptor‐ligand binding. As discussed in more detail later, the biochemical activities of the galanin receptors are dependent on the cell type in which they are expressed. Most of the effects of the galanin receptors appear to be mediated through G_i_ protein signaling, particularly through pertussis toxin‐sensitive mitogen‐activated protein kinase (MAPK) 1 and 3. Many cell types express two or all three of the receptors, and each receptor type possesses unique downstream effects. In many cases, co‐expression of galR1 and galR2 results in autoregulatory effects between the two receptors, wherein galR2 activation counteracts galR1 activation. This suggests a dynamic role in homeostatic regulation and serves to explain part of the variable findings involved in galanin signaling. Indeed, many findings have been observed wherein removing or inhibiting one receptor results in the same consequences as selective agonism of the other receptor.

## 4 Endogenous Galanin Receptor Ligands

While much of the physiological activity of the galanergic system is currently under investigation, the interactions of endogenous ligands with galanin receptors are better understood. In mammals, endogenous galanin binds all three receptor subtypes with varied affinity, with the highest affinity being for galR1, followed by galR2, and the least affinity for galR3. Human galanin has *K*_i_ values of 0.4, 2.3, and 69 nM for binding to galR1, galR2, and galR3, respectively [8] (Table 1). Other mammals show similar patterns, although with higher relative affinities for galR3 binding than humans. Porcine galanin, for example, has a *K*_i_ of 0.23 nM for galR1 and 9.8 nM for galR3 [8]. This difference is believed to be due to the additional C‐terminus serine found in human galanin, which limits its interactions with galR3. These findings come from autologous binding assays, and the affinity for galanin and receptors between species is less documented, although known to be varied [8]. That is, murine galanin does not have the same affinity for each galanin receptor across humans, pigs, and cows, and vice versa. GMAP also binds galanin receptors, with affinity dependent on splicing [11]. In rats, GMAP was found to possess affinity for galR3 but lacked significant binding to galR2 [11]. This affinity is notably less than that of galanin.

**TABLE 1:** Endogenous and recombinant galanin receptor ligands and relative receptor affinity.

Ligand	Origin	Activity	*K*_i_ (galR1) (nM)	*K*_i_ (galR2) (nM)	*K*_i_ (galR3) (nM)
Rat galanin (1–29) [8]	Endogenous	Agonist	1.0	1.5	1.5
Human galanin (1–30) [8]	Endogenous	Agonist	0.4	2.3	69
Galanin (1–16) [8]	Recombinant	Agonist	4.8	5.7	50
Galanin (2–29) [8]	Recombinant	Agonist	85	1.9	12
Galanin (3–29) [8]	Recombinant	Agonist	> 1000	> 1000	> 1000
Galanin (2–11) (AR‐M1896) [8]	Recombinant	Agonist	> 5000	88	271
Spexin (neuropeptide Q) [9]	Endogenous	Agonist	—	161^a^	626^a^
Rat GALP [8]	Endogenous	Agonist	45^a^	18.7^a^	1530^a^
Human GALP [8]	Endogenous	Agonist	77^a^	28^a^	10^a^
GMAP (1–41) [8]	Endogenous	Agonist	—	> 640	—
GMAP (44–59) [8]	Endogenous	Agonist	—	> 1000	> 1000
Alarin [8]	Endogenous	Agonist	> 1000	> 1000	> 1 000 000
[D‐Trp^2^]galanin (1–29) [10]	Recombinant	Agonist	> 1000	7.0	?

*Note:* *K*_i_ values for galR1, galR2, and galR3 are given in nM. ?: value unknown. —: no appreciable binding capacity.

^a^ Value expressed as EC50 in nM.

Spexin, also known as neuropeptide Q, is a recently discovered endogenous peptide that also preferentially binds galanin receptors. Originally identified in 2007 [12], spexin has since been implicated to have roles in metabolism, homeostasis, nociception, and reproduction [12]. The spexin gene, encoded on chromosome 12p12.1, is mostly expressed in adipose tissue, although the heart, kidney, thyroid, brain, and placental tissue also have notable expression [4]. Spexin binds galR2 with similar affinity to galanin and binds galR3 with greater affinity [13]. Interestingly, it does not bind to galR1, which may relate to its endogenous function.

### 4.1 Galanin Superfamily

**FIGURE 3:**
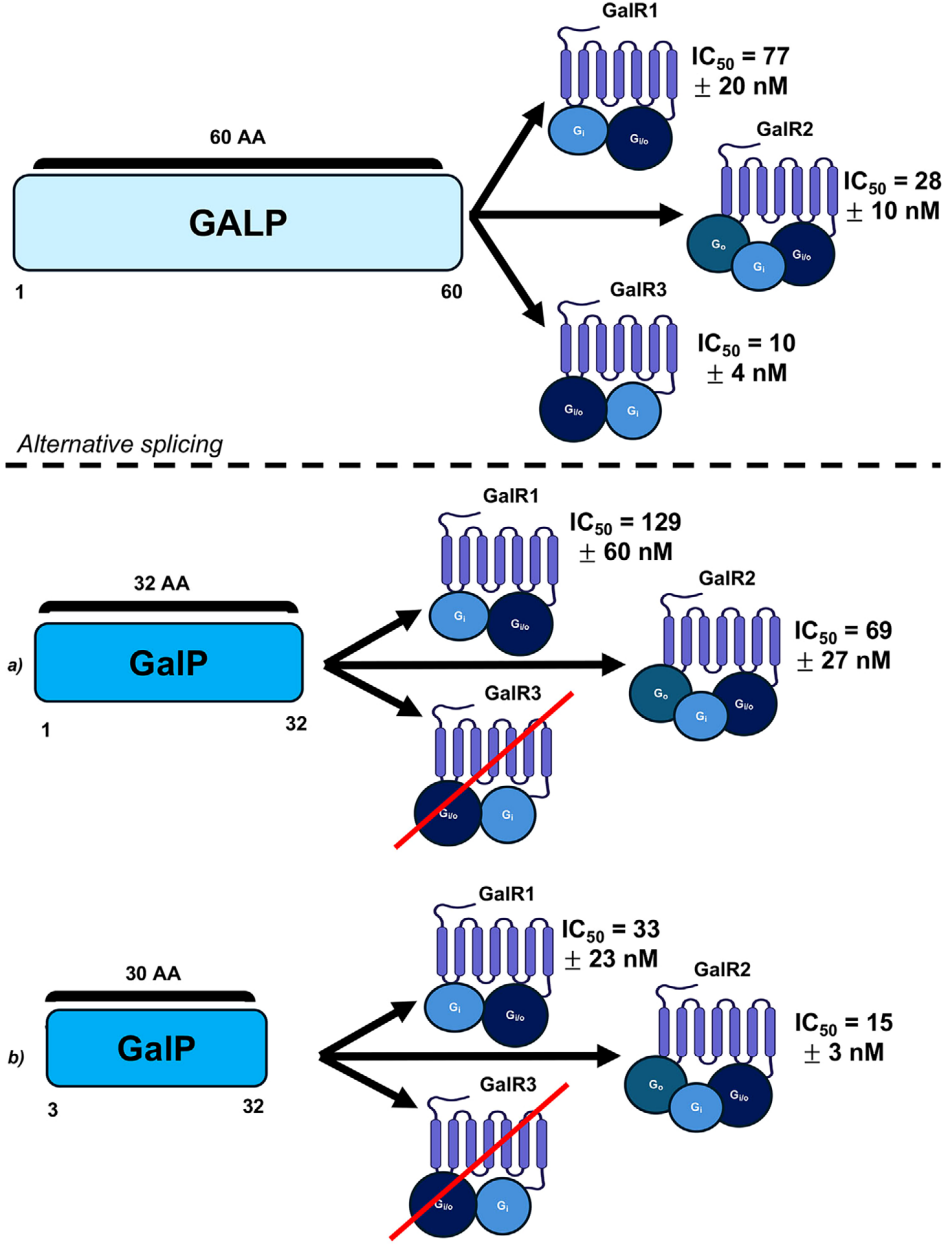
Depiction of alternative splicing of GALP. Top, endogenous GALP (1–60) and EC_50_ binding affinity to galR1, galR2, and galR3. Bottom (a) GALP (1–32) amino acids splicing and EC_50_ binding affinity to galR1, galR2. Bottom (b) GALP (3–32) amino acid splicing and EC_50_ binding affinity to galR1, galR2.

As research into galanin expands, a wider range of peptides with homology to galanin have been discovered. The galanin superfamily includes galanin‐like peptide (GALP) which has highly varied specificity dependent on posttranslational cleavage and modification of the protein, depicted in Figure 2b [14]. Figure 3 shows human GALP can bind all three receptor subtypes when uncleaved (60 amino acids long). When cleaved between the 32nd and 33rd amino acids, the shortened GALP loses galR3 binding and is a less potent binder of galR1 and galR2 [14]. When further modified to contain only amino acids 3–32, GALP remains ineffective in binding galR3 but exhibits higher affinity for binding of both galR1 and galR2 comparative to the parent protein [14]. Removing exon 3 from GALP yields alarin, a unique hormone peptide [15]. Alarin does not bind any of the three known galanin receptor subtypes [15]. Given its relation and consequential co‐expression with GALP, it is hypothesized that a unique GALP/alarin‐specific receptor exists by which these peptides enact their effect [15]. Another member of the galanin superfamily is kisspeptin. Although individually expressed and with its own receptor, kisspeptin and galanin share common genetic phylogeny [16]. While each of these molecules are fascinating in their own right, this review will focus exclusively on galanin and galanin receptor ligands.

## 5 Synthetic Galanin Receptor Ligands

**FIGURE 4:**
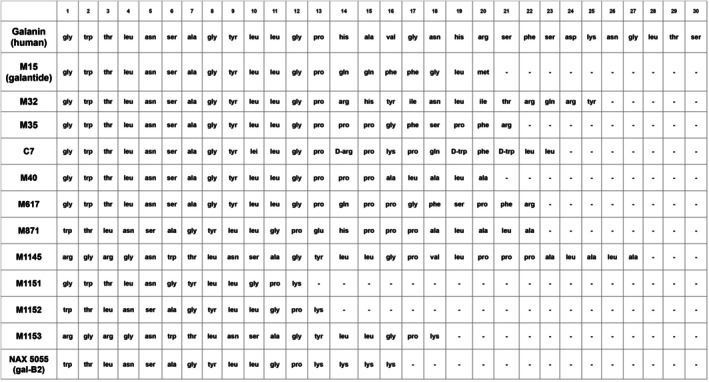
Depiction of amino acid sequences of synthetic galanin receptor ligands. Galanin (human) listed first for reference. M15, M32, M35, C7, M40, and M617 all retain the original first 13 amino acids from parent galanin. M871 and NAX 5055 retain amino acids 2–13 of galanin. Later iterations of galanin receptors ligands (M1145, M1151, M1152, M1153) also retain parts of the original 1–13 amino acid sequence of galanin, but with added amines to the beginning of the strand.

Following the discovery of the galanin receptors, a series of synthetic galanin receptor ligands were developed. M15 (galantide), M32, C7, and M40 were developed by adding amino acid chains to a cleaved 1–13 galanin fragment [17]. Figure 4 lists the amino acid sequences of peptide galanin receptor ligands. These synthetic ligands function primarily as antagonists in vivo, though at doses greater than 10 nM exhibit partial agonism at their receptors [18]. In vitro, this partial agonist property is further increased, although the mechanism behind this difference is still unclear [19]. As shown in Table 2, the various synthetics also have varied specificity for galanin receptors, which improve their utility in investigating the roles of individual receptors. M15 exhibits nonspecific binding of the three galanin receptors with slight preference for galR1, while M35 and M40 preferentially bind galR1 and 2 but still have notable binding to galR3 [8].

**TABLE 2:** Synthetic protein and non‐peptide small module galanin receptor ligands and relative receptor affinity.

Ligand	Origin	Activity	*K*_i_ (galR1) (nM)	*K*_i_ (galR2) (nM)	*K*_i_ (galR3) (nM)
M15 (galantide) [8]	Synthetic	Antagonist	0.65	1.0	1.0
M32 [20, 21]	Synthetic	Antagonist	0.26	1.45	14.1
M35 [8]	Synthetic	Antagonist	0.11	2.0	13.8
C7 [20, 21]	Synthetic	Antagonist	0.26	0.63	9.33
M40 [8]	Synthetic	Antagonist	2.4	4.1	16.2
M617 [8]	Synthetic	Agonist	0.23	5.7	49 [22]
M871 [8]	Synthetic	Antagonist	420	13	> 10 000 [22]
M1145 [8]	Synthetic	Agonist	587	6.55	497
M1151 [8]	Synthetic	Agonist	98.6	28.9	874
M1152 [8]	Synthetic	Agonist	2370	36.4	656
M1153 [8]	Synthetic	Agonist	1890	4.98	230
NAX 5055 (gal‐B2) [8]	Synthetic	Agonist	3.5	51.5	?
Galnon [8]	Non‐peptide, small molecule	agonist	11 700	34 100	?
Galmic [8]	Non‐peptide, small molecule	Agonist	34 200	> 100 000	?
Sch 202596 (spirocoumaranon) [8]	Non‐peptide, small molecule	Antagonist	1700	?	?
Dithiepine‐1,1,4,4,‐tetroxide [8]	Non‐peptide, small molecule	Antagonist	190^a^	> 30 000^a^	?
SNAP 37889 (HT‐2179) [8]	Non‐peptide, small molecule	Antagonist	> 10 000	> 10 000	17.4
SNAP 398299 [8]	Non‐peptide, small molecule	Antagonist	> 1000	> 1000	5.3
GALR3ANT [8]	Non‐peptide, small molecule	Antagonist	> 10 000	> 10 000	15

*Note:* *K*_i_ values for galR1, galR2, and galR3 given are in nM. ?: value unknown.

^a^ Value expressed as IC50 in nM.

**FIGURE 5:**
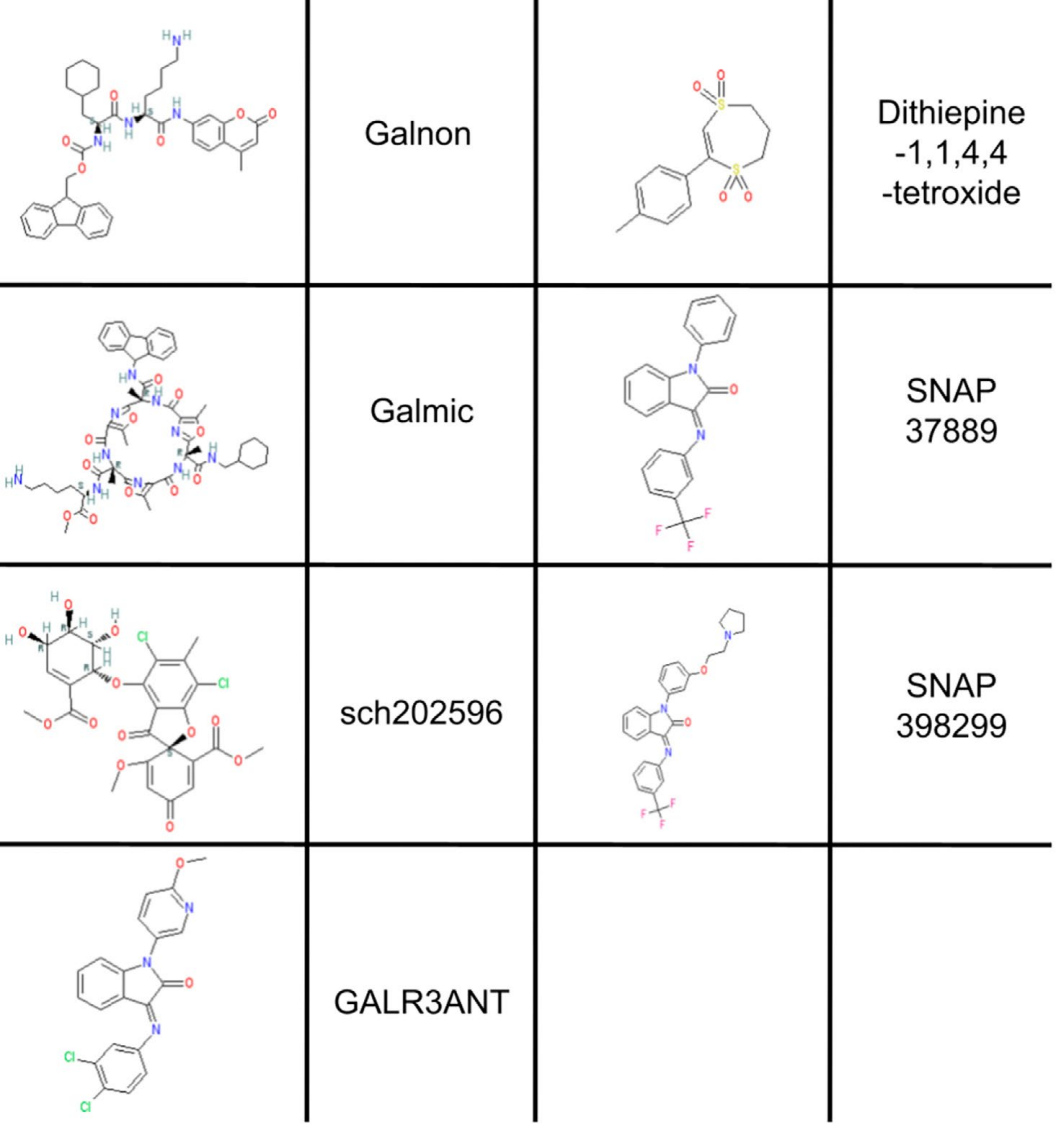
Depiction of the molecular structures of non‐peptide, small‐molecule synthetic galanin receptor ligands. Structure images provided by IUPHAR/BPS Guide to Pharmacology [24]. Galmic and galnon share similar structures, indicative of their similar origins. Similarly SNAP 37889 and SNAP 398299 share structure homology.

Following the identification of galR3, interest in studying this receptor exclusively has increased. In more recent years, galR3 selective antagonists have been developed. The use of receptor binding assays and high‐throughput screening has allowed for these newer antagonists to be small‐molecule drugs rather than peptides [23]. SNAP 27889, SNAP 398299, and GALR3ANT were all developed to be water‐soluble, highly selective galR3 antagonists [23]. Figure 5 shows the molecular structure of the current small‐molecule galanin receptor ligands. As the role of galR3 has become better understood, further questions regarding the specificity of older agents against this receptor have been prompted. In Table 2, we have collected what information is available regarding these ligands. In the case of M35 and M40, these “selective” ligands only display 4–6‐fold preference for galR1 and 2 over galR3.

An immediate challenge to exploration of these agents is their availability. Many agents, such as dithiepine‐1,1,4,4,‐tetroxide, galmic, and Sch 202596, are unavailable in the United States at the time of writing. Others, such as galnon, SNAP 37889, and M1145, only have one distributor after having been discontinued by others. Further, agents that are more widely available such as M40 or M35 have incomplete listings and fail to comment on the affinity of these agents for galR3. As a direct result of this, some investigators have been falsely led to believe these agents have no affinity for galR3, despite evidence to the contrary. Inconsistencies such as these reduce the clarity of results and may spur explorations under false pretenses while simultaneously imposing challenges in reproducibility.

## 6 Biochemical Pathways of Galanin Receptor Activation and Signaling

**FIGURE 6:**
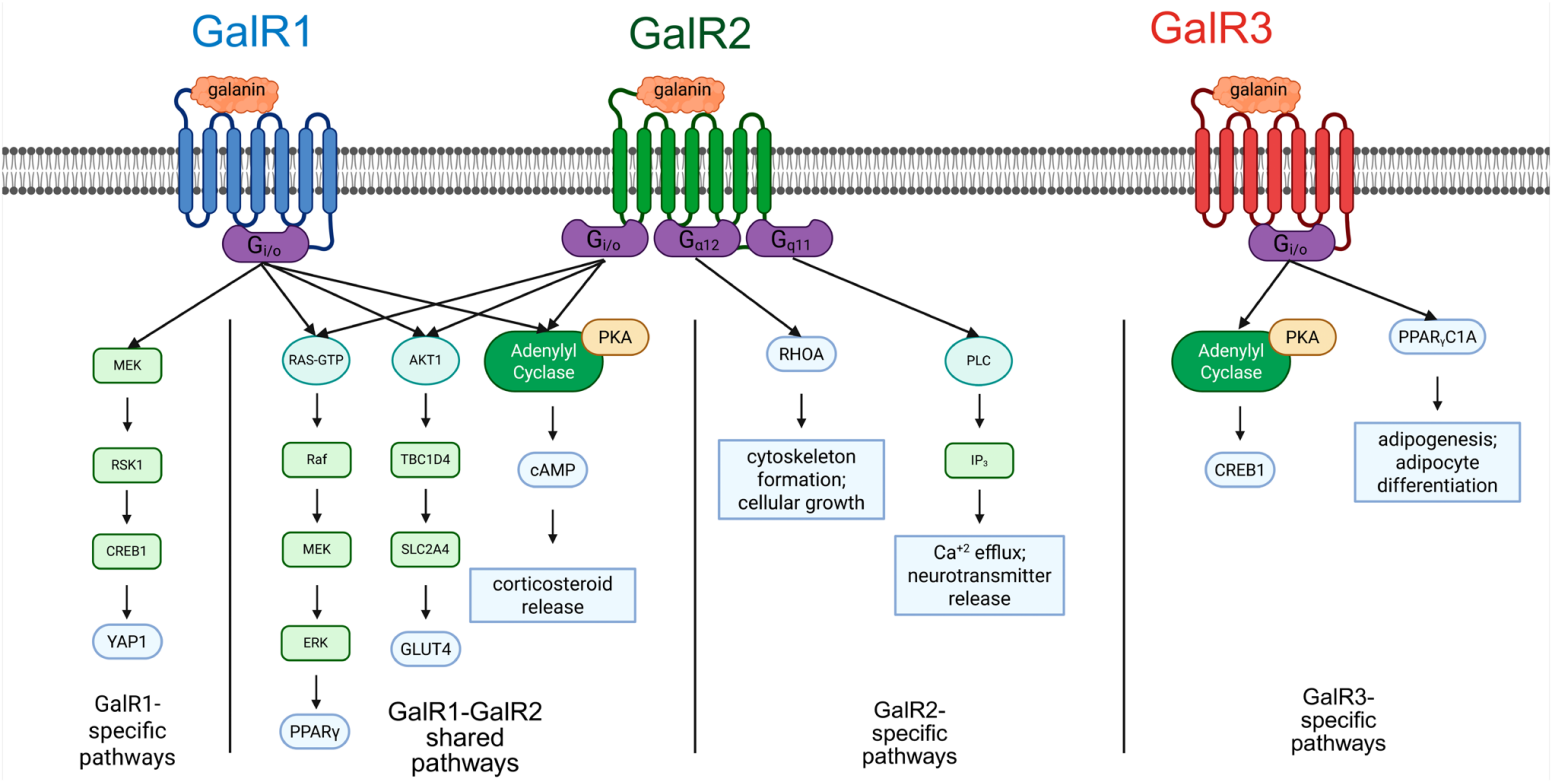
Depiction of galanin receptor signaling pathways. Left‐most: galanin receptor 1 specific pathways, middle‐left: galanin receptor 1 and galanin receptor 2 shared signaling pathways, middle‐right: galanin receptor 2 specific pathways, right‐most: galanin receptor 3 specific pathways.

To date, very few studies have elucidated the exact mechanisms by which galanin receptors exert their downstream effects. As such, most information available and presented here is observational and reports causal interactions. As a GPCR, the galanin receptors should primarily have effects related to G‐proteins, though some of their observed effects have not been linked to a G‐protein and are presumed to be direct effects. Figure 6 depicts the common biochemical pathways by which each of the activated galanin receptors exert their effects, as well as the notable overlap between galR1 and galR2 signaling. Importantly, the mechanisms by which galanin receptors interact with G‐proteins have not been discovered.

### 6.1 Galanin Receptor 1 Signaling

The known effects of galR1 agonism involve G_i/o_ activation which leads to MAPK1/3 complex phosphorylation [25]. The ultimate effects of galR1 signaling are generally lowered insulin sensitivity, increased glucose uptake, increased cellular growth and proliferation, and inhibition of apoptosis. In adipose tissue, galR1 agonism activates nuclear peroxisome proliferator‐activated receptor gamma (PPARγ) to stimulate adipocyte proliferation and differentiation [26]. PPARγ activation further leads to increases in adipokine excretion, particularly adiponectin. Mice fed a high fat diet (HFD) had increased phosphorylation of Rapidly Accelerated Fibrosarcoma proto‐oncogene serine/threonine‐protein kinase (c‐Raf) and extracellular signal‐related kinases (ERK) in their epididymal tissue compared to a normal diet [26]. The increase corresponds with the increased levels of galanin and galRs found in the same tissue. There were also increased levels of PPARγ and adaptor complex 2 (aP2), a protein complex involved in incoming vesicle transport, in this tissue [26]. The same study found decreased levels of galanin and its receptors in skeletal muscle, suggesting that galanin's role in obesity is linked to lipid absorption in adipose and reduction in glucose utilization in skeletal muscle. In subcutaneous tissue, they found increases in galanin and its receptors, as well as for ERK, PPARγ, and aP2, although they observed a dramatic decrease in c‐Raf phosphorylation [26]. The authors suggest that this finding relates to the selection of adipose depositions in HFD stimulates visceral adipogenesis and reductions in subcutaneous accumulation of lipids, and is reflective of the heterogeneity of adipose.

GalR1 receptor agonism has been found to phosphorylate rho‐family‐alpha serine/threonine protein kinase (AKT1) and subsequently stimulate glucose transporter type 4 (GLUT4) protein upregulation [27]. It was found that intracerebroventricular injection of M617, a selective galR1 agonist, to type‐II diabetic rats decreased plasma glucose and insulin levels while having increased body weights [27]. Oral glucose tolerance tests and myocyte glucose uptake testing further confirmed this effect, wherein M617 administration improved oral glucose tolerance and increased glucose uptake by myocytes. PCR and immunoblotting revealed an AKT‐dependent increase in vesicle‐associated membrane protein 2 (VAMP2), PPARγ, and GLUT4 expression in the skeletal muscle of M617‐treated mice [27]. GLUT4 increases were verified by membrane protein content, which found increased migration of GLUT4 receptors from cytoplasm to membrane [27]. The wide expression of GLUT4 thus explains many of galanin's roles involving glucose homeostasis and metabolism.

Beyond GLUT4 and PPARγ activation, galR1 signaling has been linked to a number of tissue‐specific effects. GalR1 has been shown to induce increased plasma corticosteroid excretion via protein kinase A (PKA)‐dependent adenylyl cyclase activation [28]. This function occurs primarily in the pituitary, stimulating the release of adrenocorticotropic hormone (ACTH) to induce adrenal corticosteroid release. Galanin is also implicated in the feedback regulation of corticosteroids, as it has been shown that exogenous steroid treatment greatly diminishes hypothalamic and pituitary galanin expression [29]. The precise mechanism behind this is not yet understood. Another tissue‐specific effect is in galanin's regulation of cholangiocyte proliferation. It was found that in vitro galR1 agonism of cholangiocytes upregulates ribosomal S6 kinase 1 (RSK1, also known as RPS6KA1, p90RSK) via MAPK1/3 activation to drive cyclic adenosine monophosphate responsive element binding protein 1 (CREB1) transcription of yes‐associated protein 1 (YAP1) [30]. YAP1‐mediated proliferation may support evidence of galR1's role in cancer development and proliferation.

### 6.2 Galanin Receptor 2 Signaling

GalR2 signaling shares many of the same glucose and energy metabolism pathways as galR1, but the available knowledge suggests a wider role of galR2 as the terminal effector of neuronal signaling in the periphery. GalR2 retains the ability to bind G_i/o_ subunits to drive adenylyl cyclase and PKA‐mediated cyclic adenosine monophosphate production [31]. It also shares galR1's capacity to stimulate glucocorticoid release, as well as PPARγ activation leading to GLUT4 expression [31]. GalR2 stimulation of GLUT4 has been particularly noticed in the skeletal muscle, where galR2 agonism significantly increased GLUT4, phosphorylated AKT (pAKT), PPARγ coactivator 1‐alpha (PPARγC1A), and phosphorylated AKT substrate of 160 kDa (pAS160) in HFD fed mice [32]. The overlap of galR1 and galR2 signaling further reinforces galanin's role in metabolism and may inform therapeutic strategies in targeting galanin. In contrast to galR1, galR2 has been shown to bind G_αq_ and G_12_ subunits for downstream signaling and has been more associated with effects in the cell cycle and apoptosis, particularly during fetal development [33, 34].

A healthy proportion of the knowledge regarding galR2 signaling comes from the work of Wittau et al. [34] who investigated the role of galR2 with in vitro models of small cell lung cancer (SCLC). They identified that in these cell lines only galR2 was present, and galanin agonism resulted in G_q/11_, G_a/q11_, and G_a12_ activation. Their work then investigated many of the downstream effects of the activation of these G‐protein subunits. It was found that G_q/11_ activation stimulated phospholipase C (PLC) to accumulate inositol triphosphate (IP3) and diacylglycerol (DAG) [34]. The increase in IP3 resulted in Ca^2+^ efflux from intracellular stores. It was later discovered that galR2‐mediated IP_3_ activation activates Ca^2+^‐dependent K^+^ big potassium channels responsible for neurotransmitter release [35]. Interestingly, this pathway was shown in both studies to be pertussis toxin‐insensitive, reflecting a pathway not mediated via MAPK. G_12_ stimulation resulted in Ras homolog A (RHOA) production in SCLC and galR2‐transfected fibroblast cells [34]. RHOA regulates cytoskeleton formation and facilitates cell growth and neurological development.

### 6.3 Galanin Receptor 3 Signaling

Little is known about galR3 signaling. Many studies have observed alterations in galR3 precipitate changes in metabolic responses, although these changes are often overshadowed by similar dysregulation of galR1 [26, 36, 37] or galR2 [38, 39]. As such, the exact mechanisms by which galR3 mediates its effects are broadly unknown, although it is likely that it has important roles in attenuating or assisting in the signaling pathway of the other receptors. Still, it is presumed that galR3 activation induces CREB1 and PPARγC1A activity, particularly through activation of G_i/o_ subunits.

## 7 Biologic Activities of Galanin Receptors

The wide range of galanin receptor subtypes and the varied expression of these subtypes reflect the wide and varied effects of galanin. While many systems share overlapping motifs in galanin's effects, cell types within tissue sometimes have alternating effects. This paradoxical attribute of both galanin overexpression and inhibition producing similar results suggests an impressive regulatory role of galanin in these systems. While much work remains to be done in pinpointing the roles of galanin in particular cells, its overt physiological effects are better characterized.

### 7.1 Brain and Neurological Signaling

Originally isolated as a neurotransmitter, many of galanin's physiological effects have been suspected to be related to neuronal stimulation. Galanin plays key roles in fetal and placental development, and possesses regulatory effects on growth signaling, appetite, lipid homeostasis, nociception, and anxiety [40, 41, 42, 43]. All the galR subtypes are present in the brain and are concentrated in the hypothalamus, hippocampus, locus coeruleus, and amygdala regions of the brain, allowing them to have a diverse impact on basal state signaling [44, 45].

Recently, galanin receptors have been observed to be expressed by norepinephrine (NE) neurons within the locus coeruleus (LC) in addition to the presence of galanin [46]. The LC and its NE signaling projects into other regions of the brain, allowing for its influence in memory, sleep and wakefulness, and arousal. In pathologies of mood disorders like depression, anxiety, and schizophrenia, it is thought that neuron loss within the LC or modulated NE signaling may be responsible [46]. The study by Caramia et al. sought to characterize the expression and locations of galanin and galR1 within the LC to further determine if the co‐release of NE and galanin, and galanin receptor signaling, may also play a role in these pathologies. Single‐cell RNA‐seq revealed distinct expression profiles of LC neurons expressing galanin and other neuropeptides and galR1, most being NE neurons [46]. The study also identified that overall galanin and galR1 mRNA expression was not isolated to specific subtypes of NE neurons within the LC [46]. This gives rise to the conclusion that the role of galanin within the LC may be locally recruiting NE neurons [46]. Galanin was additionally detected within tyrosine hydroxylase‐positive (Th+) cells [46]. GalR1 was seen to be expressed by gamma‐aminobutyric acid (GABA)‐ergic neurons close to the LC proper, providing more insight into the potential projection effects galanin signaling may have in this region [46]. Interestingly, reserpine, a potent, irreversible NE depleter, treatment modulated galR1 within NE neurons. Thus, the study shows evidence of galanin and galR1 playing a modulatory and co‐agonist role in NE signaling within the LC [46].

GalR1, most often expressed in the thalamus, hypothalamus, and amygdala, is associated with pain and mood disorders. Recent evidence shows that galanin system genes in the brain interact with stress signals such as childhood adversity and recent negative life events in depression‐related and anxiety‐like phenotypes [47]. Galanin dysregulation as a consequence of early life stressors in turn altered the activity of galanin receptors. It was observed that reductions in galR1 and galR3 significantly attenuated responses to these negative life events, whereas galR2 reductions worsened response [47]. Taken together, it appears that mood is influenced by a balance of depressive galR1 and galR3 influence, opposed by antidepressive galR2 signaling [47].

GalR2, located within the dentate gyrus and CA3 region of the hippocampus, is most associated with learning and memory [48]. Studies have identified it to be neuroprotective and that galanin itself modulates the survival of neurons within the hippocampus [49]. The modulatory role of galanin via galR2 signaling seems to be through affecting the glutamate‐induced activation of AKT and ERK1 [49]. The role of galR3 is not well characterized, but its expression has been localized within the hypothalamus and parts of the mid‐ and hindbrain [50]. Receptor affinity of galR3 is also lower compared to those of galR1 and galR2. Tran et al. [50] reviewed the effects of selective galR2/3 agonism with spexin. They outlined that hypothalamic neurons with galR2 and galR3 genes are upregulated by c‐Jun N‐terminal kinase (JNK) and ERK1/2 from palmitate signaling in addition to endoplasmic reticulum stress [46]. This may indicate that the expressions of these galanin receptors are upregulated, and thus galanin signaling within the hypothalamus may be increased in certain metabolic disorders and have a downstream impact on the periphery. Recent work on the role of galanin in neurodevelopment has shown that granule cell migration in the cerebellum may be modulated in part by galanin [51]. In fetal and neonatal brains, normal developmental migration of these granule cells is increased by galanin via cyclin adenosine monophosphate and Ca^2+^ pathways upon binding with galanin receptors [51]. However, this is altered upon injury via lesions by decreasing galanin levels. This results in slowed migration of granule cells and creates abnormal development of cortical layers that may result in changes to cognition [51]. Interestingly, the study found that migration was increased in areas immediately surrounding the lesion [51], indicating some compensatory change as a result of the damage, and that galanin may be therapeutic for acute neonatal brain injuries. Galanin signaling in the brain has also been very associated with appetite regulation and metabolic diseases. Extensive work has been done outlining its relation to peripheral signaling pathways such as insulin [52], but recent studies have identified further contributions from galanin. In age‐related metabolic diseases like diabetes mellitus and obesity, galanin has been observed to be obesogenic through increased insulin resistance. Galanin itself stimulates the appetite most likely through galR3 signaling in the hypothalamus [52, 53], but kisspeptin and galanin in combination have been shown to increase the uptake and expenditure of dietary glucose [53]. This appetite regulation role of galanin is further elucidated by work investigating neuropeptide crosstalk between ventromedial‐pituitary adenylate cyclase‐activating polypeptide (PACAP) and dorsomedial‐galanin in the hypothalamus of mice. PACAP in the ventromedial region of the hypothalamus was shown to play a regulatory role in galanin expression post‐fasting of mice, as knockout mice deficient in PACAP failed to display an incremental rise in galanin level after fasting [54]. It was also found that PACAP‐neurons in the hypothalamus have strong projections to hypothalamic neurons highly expressing galanin [54]. In PACAP‐deficient mice, overall galanin expression in the region was decreased; however, mice deficient in dorsomedial hypothalamic (DMH) galanin were shown to have an opposite feeding response wherein food intake during the light cycle increased and after fasting was decreased [54]. This data implies that DMH galanin may have its own regulation of appetite downstream of ventromedial‐PACAP [54]. Elevated galanin levels within the hypothalamus have also recently been identified as a potential biomarker for the consequences of type 2 diabetes mellitus (T2DM) patients [54]. A new study has shown that hypothalamic galR2 activation increases both insulin sensitivity and bone density and mass [54]. In rats with T2DM, they have elevated plasma galanin, and this is believed to contribute toward insulin resistance. Additionally, galanin secretion is thought to be regulated by T2DM and osteoporosis [54, 55], but through signaling with hypothalamic galR2 it may ameliorate facets of the diseases [54, 55].

### 7.2 Gastrointestinal Tract

Galanin and galRs expression has been shown to change in the course of gastric ulcer [56], colitis [57], injured axons in the intestine [57], intoxication with acrylamide [58], bisphenol A13, or systemic diseases, such as type I diabetes mellitus [59]. Exposure to low doses of glyphosate, the world's most widely used pesticide, altered galanin expression in intramural neurons and galanin receptors within the pig's small intestine wall [60]. Galanin immunoreactivity is present in enteric neurons located in the submucous and myenteric plexuses, as well as in fibers projecting to the gut wall [61, 62, 63]. The study investigated changes in galanin expression in porcine stomach enteric neurons following both low and high doses of acrylamide supplementation. Increased galanin‐like immunoreactivity in both myenteric and submucous plexuses was observed in all stomach fragments studied. The proportion of galanin‐expressing cell bodies that are also immunoreactive to a vasoactive intestinal peptide (VIP), neuronal nitric oxide synthase (nNOS), and cocaine‐ and amphetamine‐regulated transcript peptide (CART) increased as well [58]. In a porcine model of T2DM, prolonged high serum glucose levels can affect the chemical phenotyping of enteric neurons [59]. Galanin plays a crucial role in neuroprotection, contributing to the neural response to diverse pathological stimuli in the gastrointestinal tract. Galanin impacts gastric emptying, suppresses acid secretion, modulates peptide release [64], influences mucosal epithelial cell absorption and blood flow [65], and affects electrolyte transport through a neural pathway in the colon [66], ion transport [67, 68], and gastrointestinal motility. Galanin influences gastrointestinal motility with both stimulatory and inhibitory effects stemming from both direct myogenic actions and nerve‐mediated mechanisms involving the release of additional transmitters.

In general, galanin actions vary depending on the tissue, the species, and the experimental conditions [69, 70, 71, 72]. Galanin is present in enteric nerves lining the GI tract where it is normally involved in regulating intestinal motility by binding to the galR1 subtype expressed by smooth muscle cells [73]. Endogenous galanin activation of galR1 on enteric neurons may indirectly regulate gastrointestinal motility and secretion by influencing the release of other transmitters or modulators. galR1 plays a key role in the prejunctional neuromodulation by galanin, which inhibits substance P and acetylcholine release during the ascending excitatory reflex of intestinal peristalsis triggered by mechanical stimulation [74]. The role of galanin, galR1, and galanergic pyloric myenteric neurons in the response of pyloric wall structures to antral ulcerations is unclear [56]. Galanin enhanced lower esophageal sphincter (LES) tone and inhibited LES relaxation following nonadrenergic noncholinergic nerve stimulation via electrical field stimulation. Galanin significantly reduced the amplitude and onset latency of lower esophageal contractions by inhibiting both cholinergic and noncholinergic peristaltic components [75]. Modulating lumbar splanchnic nerve and pelvic nerve signaling in colonic and rectal regions is crucial for treating chronic visceral pain in gastrointestinal diseases [76]. Galanin significantly reduces colonic mechano‐sensitivity at harmful distending pressures and inhibits the rapid onset of mechanical hypersensitivity to inflammatory mediators via galR1 [77]. Galanin mitigated inflammatory bowel disease (IBD) in rats, but the absence of galR3 led to increased local and systemic levels of inflammatory cytokines and chemokines [78]. Galanin hypermethylation compromises its tumor suppressor role in gastric cancer development by elevating phosphorylated AKT expression [79].

### 7.3 Hepatobiliary Systems

The effects of galanin in modulating lipid metabolism and fibrotic response have been widely noted in hepatic systems. As such, its signaling has been implicated in fatty liver disease, metabolic dysfunction‐associated steatotic liver disease (MASLD, formerly NAFLD), and metabolic dysfunction‐associated steatohepatitis (MASH, formerly NASH). Hepatocytes and native hepatic stellate cells preferentially express galR2, whereas cholangiocytes and supportive tissues express galR1 [80, 81].

HFD has proven to be a useful model to observe the role of galanin in hepatic disease. HFD has been demonstrated to increase galR2 protein expression in the liver, but galR2 antagonist treatment with M871 in primary hepatocytes increased the cytotoxicity associated with palmitic oil while decreasing hepatocyte lipid accumulation [37]. Similarly, obese mice who consumed HFD for 16 weeks saw significant reversal of lipid dysregulation following 21 days of spexin treatment [82]. These mice, presumably through galR2 agonism, saw significant reduction in serum glucose and insulin, reduced total triglyceride and cholesterol content in the liver, and an increase toward normal controls in PPARγC1A, peroxisome‐proliferator‐activated receptor alpha (PPARα), carnitine palmitoyltransferase 1A (CPT1A), and sterol regulatory element binding protein‐1c (SREBP1C) [82]. Many of these observations were reversed with the addition of M871 to spexin treatment.

In direct contrast, galR2 inhibition via M871 has also been shown to reduce liver fibrosis [80]. This study was completed in multidrug resistance‐associated protein 2 knockout (Mdr2KO) mice: a transgenic mouse model with impaired phospholipid secretion to bile and naturally develops profound liver fibrosis. In this non‐obesogenic model of hepatic inflammation and cholestasis, galR1 depletion via galR1 vivo morpholino significantly reduced cholangiocyte proliferation markers cytokeratin 19 (CK‐19) and proliferating cell nuclear antigen (PCNA) expression and decreased fibrotic markers alpha‐smooth muscle actin (α‐SMA) and collagen type 1 alpha 1 (Col1A1) [80]. In contrast, M871 antagonism of galR2 did not significantly alter CK‐19 expression, but resulted in significant reductions in desmin, α‐SMA, and Col1A1 expression, while also greatly reducing lipid accumulation as shown by sirius red staining [80]. Finally, joint inhibition of galR1 and 2 via M40 attenuated both biliary and fibrotic responses, showing recovery in most markers [80]. These findings could suggest that early inhibition of galR2 is protective against fibrosis, while following lipid accumulation and progression of fatty liver disease galR2 agonism is beneficial.

Specific interest has been placed in observing the role of galanin in biliary disease. Spexin has been demonstrated to induce changes to both bile acid secretion and content [39]. One hour post spexin administration resulted in reduced bile acid concentration in plasma and liver and modest increases in intestinal luminal bile acid content [39]. Chronic, 28‐day administration resulted in significant, dose‐dependent reductions in serum triglyceride and bile acid concentrations, body weight, and gallbladder weight. Chronic administration also significantly altered bile acid contents of the liver, gallbladder, and ileum, particularly tauro‐β‐muricholic acid, tauroursodeoxycholic acid, glycocholic acid, and cholic acid [39]. Galanin has also been shown to impact cholestatic disease in an obstructive model of bile duct ligation (BDL). Rats 3‐ and 7‐days post BDL surgery showed significant increases in cholangiocyte expression of galanin in addition to their marked disease burden [30]. Further treatment with recombinant galanin increased the bile duct mass and proportion of reactive cholangiocytes in both normal (sham) and BDL rats compared to matched controls. In vitro, a cholangiocyte cell line displayed a dose‐dependent increase in proliferation via MTS assay [30]. These results were further confirmed to be the result of galR1 activation as similar findings were produced using M617 selective agonism of galR1. Following this, circulating galanin was reduced using a galanin vivo morpholino which resulted in reductions in ductular reactions via CK‐19 and PCNA expression as well as reduced bile duct mass in BDL rats [30].

### 7.4 Endocrine Signaling and Adipose

Galanin‐mediated signaling pathways are recognized for their influence on adipogenesis [83]. Earlier studies indicate that chronic administration of galanin into the paraventricular nucleus of the hypothalamus leads to intricate alterations in daily caloric intake, levels of obesity, and regional fat deposition, depending on the fat and carbohydrate content of the diet [84]. The rats fed HFD displayed 40% higher hypothalamic galanin levels [85]. Also, the mice fed an HFD displayed higher mRNA levels of galanin in epididymal adipose tissues along with increased expression of galR1, galR2, and galR3 gene levels in both their epididymal and subcutaneous adipose tissues [26]. Extended HFD also activates the galanin‐mediated signaling molecules, such as Ras/c‐Raf/MEK/ERK (via galR1) and protein kinase C delta (PKCδ)/ERK (via galR2) pathways in the epididymal adipose tissues of mice [26]. Unlike epididymal adipose tissues, subcutaneous fat tissue in both mouse groups showed no difference in Ras mRNA levels, and phosphorylated c‐Raf was significantly decreased by HFD feeding, indicating that the HFD‐induced activation of galanin‐mediated adipogenesis signaling occurs via the galR2/PKCδ/ERK pathway in the subcutaneous adipose tissue. Galanin significantly influences adipogenesis by modulating the expression of PPARγ2, a nuclear receptor subfamily transcription factor involved in regulating genes critical for adipocyte differentiation [86]. Galanin synthesis is associated with fatty acid metabolism because blocking fatty acid metabolism with mercapto‐acetate reduces the galanin expression in the anterior paraventricular nucleus [87]. Some natural compounds were found to target galanin to regulate adipogenesis. Oleuropein, a natural polyphenolic compound belonging to the secoiridoids, significantly decreases HFD‐induced body weight gain and visceral adiposity via downregulation of genes involved in galanin‐mediated signaling cascades [83]. Cinchonine, a natural compound of cinchona bark, regulates adipogenesis by significantly reversing the HFD‐induced increases of adipogenic genes involved in galanin‐mediated signaling pathways, markedly downregulated galRs and consequently reduced the expression of PPARγ2 and its target genes (Ca/Enhancer‐binding protein α, leptin, aP2, and lipoprotein lipase) [86]. Carvacrol, a monoterpene phenolic constituent of essential oil from aromatic plants and spices, inhibits visceral adipogenesis probably by suppressing galanin‐mediated signaling [88].

Adipose tissue functions not only as a fat depot but also as a significant endocrine organ, synthesizing and secreting various factors. Leptin, an adipocyte‐derived hormone, is crucial for body weight and energy balance regulation, functioning through hypothalamus receptors in rodents to inhibit feeding and increase thermogenesis [89, 90]. Leptin, “the voice” of adipose tissue, communicates the status of peripheral lipid reserves to the brain [90]. The brain then regulates food intake and fat stores by coordinating various hypothalamic peptide systems, including neuropeptide Y and galanin [91]. Leptin synthesis and secretion are regulated by a complex interplay of neuroendocrine, endocrine, and paracrine signals [92]. Galanin inhibits leptin production in vivo and in vitro, and the increased galanin expression in rat adipose tissue after fasting may regulate leptin expression and secretion in fasting rats [92]. Galanin modulates the potency of leptin signals that modify food intake in the neonatal rat by interacting with leptin at the level of the stomach to decrease afferent neuronal signals to the nucleus tractus solitarius [93]. The paraventricular nucleus, a crucial area for leptin's impact on food intake [94], is also vital for the effects of galanin on food intake and body weight, and levels of galanin peptide and gene expression in this region correlate with natural feeding preferences [95]. Galanin peptide stimulates fatty acid β‐oxidation in the liver and lipolysis in adipose tissue, indicating that the anti‐obesity effect of galanin peptide may stem from anorexigenic actions and improvement of lipid metabolism in peripheral tissues via the sympathetic nervous system [96]. Overall, elevated circulating galanin levels play a role in metabolic syndrome development, with potential therapeutic effects of galanin and its receptor ligands on fat deposition and lipid metabolism [97].

Moreover, leptin may activate the sympathetic nervous system and thermogenesis through a melanocortin‐independent mechanism involving galanin peptide [98]. Galanin plays a role in thermoregulatory behaviors and metabolism [99]. Intracerebroventricular administration of galanin‐like peptide, which is upregulated by leptin and thus could participate in leptin‐mediated stimulation of brown adipose tissue (BAT) activity, enhances BAT thermogenesis in a melanocortin‐independent manner [100]. Increased galRs expression in the expanded adipose tissue suppresses the thermogenesis‐signaling pathways [26] by inhibiting forskolin‐stimulated cyclic adenosine monophosphate production in a pertussis toxin‐sensitive manner leading to reduced CREB activation [48].

### 7.5 Micro‐ and Cardio‐Vascular Systems

Discovery of the wider range of endogenous galanin ligands, as well as interest in galR3, has uncovered wider roles of galanin in the skin and vasculature. GALP, GMAP, and spexin have all been shown to be expressed widely in these systems, and their effects have been slowly elucidated [101]. Galanin administration significantly reduced murine edema in response to substance P + calcitonin gene‐related peptide (CGRP)‐induced inflammation [101, 102]. Further exploration using galanin [2, 3, 4, 5, 6, 7, 8, 11, 12, 13], SNAP 37889, and GALP revealed similar effects, wherein selective galR2 and galR3 activation reduced edema, and inhibition of galR3 signaling via SNAP 37889 abolished galanin's effects [102]. Thus, while the mechanism is uncertain, galR3 signaling has a clear effect in inhibiting histamine‐induced angioedema [102]. It is also suggested that galanin signaling counteracts the vasodilatory effects of peripheral neuropeptide Y and CGRP [103].

In regard to cardiac health, spexin has attracted attention. In models of hypoxic and doxorubicin‐induced cardiomyocyte damage, spexin has demonstrated protective effects against these conditions [104, 105]. Spexin activation of galR3 was demonstrated to mitigate reactive oxygen species damage during hypoxic conditions, an effect which remained even in the presence of the selective galR2 antagonist M871 [104]. Similarly, spexin protected cardiac function against doxorubicin damage in both acute and chronic in vivo models [105]. This was further supported by in vitro experiments in which spexin reduced doxorubicin‐induced cardiomyocyte death [105]. It was further explored and discovered that spexin mediates ferroptotic responses being induced by doxorubicin toxicity, and spexin was also protective against classical erastin‐induced ferroptosis in vitro. As a neurotransmitter, spexin also exerts an effect on cardiac conductance via Ca^2+^ transport homeostasis [106]. In a spexin knockout mouse line, arterial cardiomyocytes had impaired calcium homeostasis, although ventricular cardiomyocytes did not have an impairment [106]. This change did not induce atrial fibrillation, but by altering calcium levels does increase susceptibility to developing atrial fibrillation [106]. Knocking out galR2 in these mice produced the same effect, implicating spexin's agonism of galR2 as the main mechanism behind these observations. Finally, mice that underwent angiotensin‐II treatment for the induction of atrial fibrillation were protected by concurrent treatment with exogenous spexin. Thus, while a loss of spexin expression increases vulnerability to developing atrial fibrillation, treatment with spexin is protective in mice [106].

### 7.6 Reproductive System

Terminal projections of the peripheral nervous system in both the male and female reproductive systems have been found to regulate various physiological functions through galanin signaling [107, 108, 109, 110]. Investigations into galanin's role in genitourinary health include development, reproductive health, nonreproductive physiology, and maternal health.

In the female reproductive organs, galanin has been shown to influence uterine contractility, and relationships between galanin and polycystic ovarian syndrome and pregnancy have been explored [107, 108]. The study found *Escherichia coli* aggravation of uterine tissue substantially lowered galR1 expression via immunofluorescence and western blot, but did not change galR2 expression [107]. Direct galanin treatments at 10^−8^ and 10^−7^ M reduced the amplitude of contractions of inflamed uteri compared to controls and increased the frequency of myometrial contractions of inflamed uteri compared to controls [107]. This study suggests a role of galanin signaling in uterine contractions, particularly in response to inflammatory signaling. Clinically, reduced amplitude of contractility increases inflammatory sediment exposure of uterine tissue, encouraging continued inflammatory response, and in turn, the increased frequency encourages removal of debris from the cavity to a limited extent [107].

Another role of the galanin system in regulating response to pathogens stems from its intrinsic fungistatic properties against many various *Candida* strains normally present in the female reproductive tract. In a study from Rauch et al. [111], GMAP was shown to inhibit growth and differentiation of several strains of *Candida albicans* . Although the mixture of GMAP and galanin was not able to inhibit *Escherichia coli* , *Staphylococcus aureus* , or *Candida jeikeium* growth, it was able to prevent yeast‐to‐hyphal transformations in *Candida albicans* in a dose‐dependent manner [111]. This discovery adds the galanin system to the list of endogenous antimicrobial peptides. Further studies revealed efficacy of GMAP in *glabrata*, *krusei*, *lusitaniae*, *parapsilosis*, *pelliculosa*, *tropicalis*, and *utilis* Candida strains [111]. Thus, in a larger picture, increases in preprogalanin may stimulate inflammatory responses from galanin while helping defend against fungal pathogens, implicating a multimodal mechanism of galanin systems in innate immunity, particularly in maintaining vaginal microbiome homeostasis.

In the male reproductive system, galanergic signaling has been implicated in innervation relating to control of erectile function. In a study by Yuan et al. [110], it was observed that castrated rats had markedly reduced galanin receptor and galanin expression in penile epithelial tissue. As expected, castration impaired erectile responses in these mice, which were improved following testosterone treatment. Rats transfected with a galanin overexpressing vector had improved erectile function after castration in the absence of testosterone treatment [110]. Western blot analysis showed castration increased rho‐associated coiled‐core kinase (ROCK) 1 and 2 expression while reducing the activation of endothelial nitric oxide synthase. Both testosterone and galanin treatment groups showed a recovery in these markers. This study suggests galanin's role in mediating the erectile response in a nitric oxide dependent, testosterone independent mechanism [110].

### 7.7 Growth and Development

Galanin's role in embryonic development is complicated and has yet to be extensively explored. The importance of galanin receptors in neurological development has been especially noted in the growth in the forebrain and in many terminal synapses involved in olfactory and pain sensory [112]. It has been shown that galanin applied to in vitro primary striatal neuroprogenitor cells increased B‐cell lymphoma‐extra large (Bcl‐xL) and B‐cell lymphoma‐2 (Bcl‐2) expression, and in galanin‐knockout cells also increased mammalian Achaete‐Scute homolog‐1 (MASH‐1) and oligodendrocyte transcription factor 2 (OLIG2) [112]. Important to note is that these neuroprogenitor cells expressed galR1 and galR2 but not galR3, and the study did not isolate which receptor specifically was responsible for these changes. Galanin stimulation of these genes increased cell proliferation shown by MTT assay, although no relation to neuronal differentiation was observed [112]. Further, despite the clear effect of galanin on neuronal proliferation, galanin‐knockout mice did not show impairment to olfactory capability despite diminished proliferating neurons, reflecting galanin's important but not exclusive role in neurogenesis [112].

Work from Aisha El‐Bareg has elucidated much of the role of galanin in embryonic development [113]. Experiments with in vitro human embryonic stem cells (hESCs) revealed that galanin is highly expressed along with many other pluripotent cell markers like octamer‐binding transcription factor 4 (OCT4) and NANOG [113]. Similar to findings from neuroprogenitor cells, when differentiated with retinoic acid, these hESCs lose significant galanin expression. This reinforces the hypothesis that galanin is a crucial factor in proliferation and maintenance of pluripotency in stem cells, but is not critical for differentiation.

Galanin's role in energy homeostasis has also warranted the investigation of its role in gestational diabetes, a common pregnancy complication. In a retrospective human study in China, it was found that circulating galanin was positively correlated with prepregnancy body mass index and weight of women with gestational diabetes [114]. Given galanin's roles in fetal development, this correlation could suggest a mechanism of gestational diabetes and fetal abnormalities, but the extent to which circulating galanin crosses the placenta has yet to be measured. Placental expression of galanin has been widely noted, though expression levels vary drastically with regard to gestational age [115]. Umbilical cord galanin was found to be positively correlated with birth weight, although the primary source and expression pattern of umbilical cord galanin is not completely understood [116]. A reasonable hypothesis is that fetal galanin production stimulates weight growth and is responsible for the observed change. Less clear is the extent to which maternal circulating galanin influences fetal or placental galanin secretion, as the placenta is currently presumed to be the main maternal source of fetal galanin.

### 7.8 Oncology

Galanin's role in cellular growth and replication has naturally drawn interest in exploring its role in cancer. Galanin may have a role in certain cancers of the brain, as a new study investigated galanin and galanin receptor expression in pituitary adenoma and glioma patients. Using human tumor sections, they were able to find the expression of galanin and all 3 galanin receptors (to varying degrees) in different tumors of the brain [117]. mRNA and immunohistochemical detection were utilized for this approach, but it validates other work performed in mouse studies of galanin in similar tumor types [118]. Tumors of the pituitary appear to express increased galR1 while galR3 expression only increased in recently relapsed remission patients [118]. Interestingly, galR2 does not appear to be expressed by tumors, indicating its role is less beneficial for tumors [118]. Galanin‐expressing cells within these tumors also appear to be co‐expressing other key regulatory hormones such as ACTH, growth hormone (GH), and prolactin [118].

Other studies have explored the relationship between galanin and cholangiocarcinoma. Immunohistochemical staining of human samples from healthy tissue and biopsy from patients with perihilar cholangiocarcinoma (pCCA) [81]. Cholangiocytes were found to highly express galanin with no variation in cancer state. GalR1 was also found, and although there were modest increases in galR1 expression in pCCA patients with cholestasis, significance was not reached. Conversely, while little expression of galR2 was present in the normal state, there were significant reductions in pCCA patients with cholestasis [81]. GalR3 was lowly expressed in cholangiocytes, and as such, little variance was observed. The same pattern was observed when comparing the expressions of tumor cells [81]. A correlation was also observed between high galanin expression and prolonged survival in patients, and between lower expression of galR3 and prolonged survival, though neither met significance. The same group explored spexin expression in the same groups and found no significant difference in spexin expression between groups, although large variance was observed within peritumoral and pCCA groups [119]. Clinically, a correlation between high spexin expression and prolonged patient survival was observed, although it did not achieve significance.

Beyond relations between galanin and tumors, few studies have been completed targeting galanin in tumors. One study, however, has identified that alterations of galR1 in colorectal cancer samples were correlated to patient response to 5‐fluorouracil and oxaliplatin chemotherapy [120]. Along with many other markers, it was observed that patients with lower expression of galR1 were more likely to have progression‐free survival than high‐expressing counterparts [120]. This relationship was further explored in vitro, where inhibition of galanin or galR1 via siRNA in human colorectal carcinoma cells significantly increased the apoptotic lethality of 5‐fluorouracil, oxaliplatin, and the combination [120]. Finally, they observed the effect was due to galR1's effects in mediating caspase, as silencing galR1 in the presence of the pan‐caspase inhibitor Z‐Val‐Ala‐Asp‐fluoromethylketone (zVAD) attenuated the observed increase in chemotherapy toxicity [120]. Taken together, this shows galR1 may be preferentially upregulated in some cancer types to benefit from its antiapoptotic effects on caspase activation, and addition of galR1 antagonists may prove beneficial as adjunctive therapy.

## 8 Future Directions

While a wealth of ever‐growing knowledge surrounding galanin has been collected in the past decades, an ever‐growing field of questions has been uncovered in the process. Perhaps the greatest deficit in our understanding is the lack of knowledge of galanin's kinetics and dynamics. As galanin's effects are found across the body of all of the animals studied, there is a growing need to understand how galanin ligands circulate through the body and potentially carry wide, off‐target effects. As an example, circulating galR1 inhibitors may alter insulin sensitivity in muscles, but might also alter satiety centrally. This information will also further the potential of these galanin receptor ligands as therapeutic agents.

As is the case in most fields of research, greater collaboration is necessary, but this is especially true for galanin. Many of the references provided here attempt to follow observations from other groups and often find counterintuitive effects in their field. The fine balance in galR1 and galR2 expression leads to conflicting results when one receptor type is targeted over the other, and the effects appear to be varied depending on the tissue type. Greater attention to detail in recognizing the presence of all three receptor subtypes in samples, as well as which receptors are being affected via testing, remains critical to mitigating these inconsistencies. As previously mentioned, better reporting from distributors regarding galanin ligands will further improve receptor targeting, and wider production of these ligands will increase access to these valuable tools and improve the ability of investigators to reproduce results found.

## Author Contributions

**Patrick Mireles:** conceptualized the manuscript (lead), writing – original draft preparation (lead), writing – reviewing and editing (equal). **Yubo Wang:** writing – original draft preparation (supporting), writing – reviewing and editing (equal). **Kathryn Rhodes:** writing – original draft preparation (supporting), writing – reviewing and editing (equal). **Sharon DeMorrow:** conceptualization (supporting), writing – original draft (supporting), writing – review and editing (equal).

## Funding

This study was funded by NIH R01 awards (DK135995) to Sharon DeMorrow. The funders had no role in study design, data collection and analysis, decision to publish, or preparation of the manuscript.

## Conflicts of Interest

The authors declare no conflicts of interest.

## Data Availability

No new data. Data sharing is not applicable to this article as no datasets were generated or analyzed during the current study.

## References

[1] K. Tatemoto , A. Rokaeus , H. Jornvall , T. J. McDonald , and V. Mutt , “Galanin—A Novel Biologically Active Peptide From Porcine Intestine,” FEBS Letters 164 (1983): 124–128. 6197320 10.1016/0014-5793(83)80033-7

[2] K. Kask , U. Langel , and T. Bartfai , “Galanin—A Neuropeptide With Inhibitory Actions,” Cellular and Molecular Neurobiology 15 (1995): 653–673. 8719035 10.1007/BF02071130 PMC11563080

[3] H. Evans , M. Baumgartner , J. Shine , and H. Herzog , “Genomic Organization and Localization of the Gene Encoding Human Preprogalanin,” Genomics 18 (1993): 473–477. 7508413 10.1016/s0888-7543(11)80002-9

[4] N. A. O'Leary , E. Cox , J. B. Holmes , et al., “Exploring and Retrieving Sequence and Metadata for Species Across the Tree of Life With NCBI Datasets,” Scientific Data 11 (2024): 732. 38969627 10.1038/s41597-024-03571-y PMC11226681

[5] S. Kowalyk , R. Veith , M. Boyle , and G. J. Taborsky, Jr. , “Liver Releases Galanin During Sympathetic Nerve Stimulation,” American Journal of Physiology 262 (1992): E671–E678. 1375437 10.1152/ajpendo.1992.262.5.E671

[6] L. Zhang , W. Yu , I. Schroedter , J. Kong , and M. Vrontakis , “Galanin Transgenic Mice With Elevated Circulating Galanin Levels Alleviate Demyelination in a Cuprizone‐Induced MS Mouse Model,” PLoS One 7 (2012): e33901. 22442732 10.1371/journal.pone.0033901 PMC3307774

[7] H. Harling and J. J. Holst , “Circulating Galanin: Origin, Metabolism, and Pharmacokinetics in Anesthetized Pigs,” American Journal of Physiology 262 (1992): E52–E57. 1370746 10.1152/ajpendo.1992.262.1.E52

[8] K. E. Webling , J. Runesson , T. Bartfai , and U. Langel , “Galanin Receptors and Ligands,” Frontiers in Endocrinology 3 (2012): 146. 23233848 10.3389/fendo.2012.00146 PMC3516677

[9] D. K. Kim , S. Yun , G. H. Son , et al., “Coevolution of the Spexin/Galanin/Kisspeptin Family: Spexin Activates Galanin Receptor Type II and III,” Endocrinology 155 (2014): 1864–1873. 24517231 10.1210/en.2013-2106

[10] K. E. Smith , C. Forray , M. W. Walker , et al., “Expression Cloning of a Rat Hypothalamic Galanin Receptor Coupled to Phosphoinositide Turnover,” Journal of Biological Chemistry 272 (1997): 24612–24616. 9305929 10.1074/jbc.272.39.24612

[11] S. Wang , T. Hashemi , C. He , C. Strader , and M. Bayne , “Molecular Cloning and Pharmacological Characterization of a New Galanin Receptor Subtype,” Molecular Pharmacology 52 (1997): 337–343. 9281594 10.1124/mol.52.3.337

[12] O. Mirabeau , E. Perlas , C. Severini , et al., “Identification of Novel Peptide Hormones in the Human Proteome by Hidden Markov Model Screening,” Genome Research 17 (2007): 320–327. 17284679 10.1101/gr.5755407 PMC1800923

[13] A. Reyes‐Alcaraz , Y. N. Lee , G. H. Son , et al., “Development of Spexin‐Based Human Galanin Receptor Type II‐Specific Agonists With Increased Stability in Serum and Anxiolytic Effect in Mice,” Scientific Reports 6 (2016): 21453. 26907960 10.1038/srep21453 PMC4764904

[14] R. Lang , A. Berger , R. Santic , et al., “Pharmacological and Functional Characterization of Galanin‐Like Peptide Fragments as Potent Galanin Receptor Agonists,” Neuropeptides 39 (2005): 179–184. 15944009 10.1016/j.npep.2004.12.015

[15] C. K. Boughton , M. Patterson , G. A. Bewick , et al., “Alarin Stimulates Food Intake and Gonadotrophin Release in Male Rats,” British Journal of Pharmacology 161 (2010): 601–613. 20880399 10.1111/j.1476-5381.2010.00893.x PMC2990158

[16] L. Frerichs , N. R. Smith , K. H. Lich , T. K. BenDor , and K. R. Evenson , “A Scoping Review of Simulation Modeling in Built Environment and Physical Activity Research: Current Status, Gaps, and Future Directions for Improving Translation,” Health & Place 57 (2019): 122–130. 31028948 10.1016/j.healthplace.2019.04.001 PMC6589124

[17] K. E. Smith , M. W. Walker , R. Artymyshyn , et al., “Cloned Human and Rat Galanin GALR3 Receptors. Pharmacology and Activation of G‐Protein Inwardly Rectifying K+ Channels,” Journal of Biological Chemistry 273 (1998): 23321–23326. 9722565 10.1074/jbc.273.36.23321

[18] G. Barreda‐Gomez , I. Manuel , and R. Rodriguez‐Puertas , “Neuroanatomical Characterization of the G Protein‐Coupled Receptor Activity Evoked by Galanin‐Related Ligands,” Journal of Chemical Neuroanatomy 128 (2023): 102226. 36566994 10.1016/j.jchemneu.2022.102226

[19] L. W. Fitzgerald , J. P. Patterson , D. S. Conklin , R. Horlick , and B. L. Largent , “Pharmacological and Biochemical Characterization of a Recombinant Human Galanin GALR1 Receptor: Agonist Character of Chimeric Galanin Peptides,” Journal of Pharmacology and Experimental Therapeutics 287 (1998): 448–456. 9808667

[20] C. R. Robertson , E. A. Scholl , T. H. Pruess , B. R. Green , H. S. White , and G. Bulaj , “Engineering Galanin Analogues That Discriminate Between GalR1 and GalR2 Receptor Subtypes and Exhibit Anticonvulsant Activity Following Systemic Delivery,” Journal of Medicinal Chemistry 53 (2010): 1871–1875. 20121116 10.1021/jm9018349 PMC2846716

[21] B. Borowsky , M. W. Walker , L. Y. Huang , et al., “Cloning and Characterization of the Human Galanin GALR2 Receptor,” Peptides 19 (1998): 1771–1781. 9880084 10.1016/s0196-9781(98)00133-8

[22] U. E. Sollenberg , J. Runesson , R. Sillard , and Ü. Langel , “Binding of Chimeric Peptides M617 and M871 to Galanin Receptor Type 3 Reveals Characteristics of Galanin Receptor–Ligand Interaction,” International Journal of Peptide Research and Therapeutics 16 (2010): 17–22.

[23] M. J. Konkel , M. Packiarajan , H. Chen , et al., “Amino Substituted Analogs of 1‐Phenyl‐3‐Phenylimino‐2‐Indolones With Potent Galanin Gal3 Receptor Binding Affinity and Improved Solubility,” Bioorganic & Medicinal Chemistry Letters 16 (2006): 3950–3954. 16730981 10.1016/j.bmcl.2006.05.025

[24] S. D. Harding , J. F. Armstrong , E. Faccenda , et al., “The IUPHAR/BPS Guide to PHARMACOLOGY in 2026,” Nucleic Acids Research (2025): gkaf1067, 10.1093/nar/gkaf1067. 41160876 PMC12807616

[25] I. Martinelli , A. Timotin , P. Moreno‐Corchado , et al., “Galanin Promotes Autophagy and Alleviates Apoptosis in the Hypertrophied Heart Through FoxO1 Pathway,” Redox Biology 40 (2021): 101866. 33493902 10.1016/j.redox.2021.101866 PMC7823211

[26] A. Kim and T. Park , “Diet‐Induced Obesity Regulates the Galanin‐Mediated Signaling Cascade in the Adipose Tissue of Mice,” Molecular Nutrition & Food Research 54 (2010): 1361–1370. 20183829 10.1002/mnfr.200900317

[27] L. Bu , X. Chang , X. Cheng , et al., “Activated Central Galanin Type 1 Receptor Alleviated Insulin Resistance in Diabetic Rat Muscle,” Journal of Neuroscience Research 94 (2016): 947–955. 27410235 10.1002/jnr.23775

[28] C. Tortorella , G. Neri , and G. G. Nussdorfer , “Galanin in the Regulation of the Hypothalamic‐Pituitary‐Adrenal Axis (Review),” International Journal of Molecular Medicine 19 (2007): 639–647. 17334639

[29] R. S. Brogan , L. K. Coney , W. B. Wehrenberg , G. Beretta , and A. Giustina , “Short‐Term Glucocorticoid Administration Decreases Both Hypothalamic and Pituitary Galanin Synthesis in the Adult Male Rat,” Metabolism 48 (1999): 792–796. 10381156 10.1016/s0026-0495(99)90181-6

[30] M. McMillin , G. Frampton , S. Grant , and S. DeMorrow , “The Neuropeptide Galanin Is Up‐Regulated During Cholestasis and Contributes to Cholangiocyte Proliferation,” American Journal of Pathology 187 (2017): 819–830. 28196718 10.1016/j.ajpath.2016.12.015 PMC5397710

[31] P. G. Andreis , L. K. Malendowicz , P. Rebuffat , R. Spinazzi , A. Ziolkowska , and G. G. Nussdorfer , “Galanin Enhances Corticosterone Secretion From Dispersed Rat Adrenocortical Cells Through the Activation of GAL‐R1 and GAL‐R2 Receptors Coupled to the Adenylate Cyclase‐Dependent Signaling Cascade,” International Journal of Molecular Medicine 19 (2007): 149–155. 17143559

[32] P. Fang , L. Zhang , M. Yu , et al., “Activiated Galanin Receptor 2 Attenuates Insulin Resistance in Skeletal Muscle of Obese Mice,” Peptides 99 (2018): 92–98. 29183756 10.1016/j.peptides.2017.11.018

[33] W. Jiang and S. Zheng , “Structural Insights Into Galanin Receptor Signaling,” Proceedings of the National Academy of Sciences of the United States of America 119 (2022): e2121465119. 35594396 10.1073/pnas.2121465119 PMC9173784

[34] N. Wittau , R. Grosse , F. Kalkbrenner , A. Gohla , G. Schultz , and T. Gudermann , “The Galanin Receptor Type 2 Initiates Multiple Signaling Pathways in Small Cell Lung Cancer Cells by Coupling to G(q), G(i) and G(12) Proteins,” Oncogene 19 (2000): 4199–4209. 10980593 10.1038/sj.onc.1203777

[35] N. C. Pan , Y. F. Bai , Y. Yang , T. Hokfelt , and Z. Q. Xu , “Activation of Galanin Receptor 2 Stimulates Large Conductance Ca(2+)‐Dependent K(+) (BK) Channels Through the IP3 Pathway in Human Embryonic Kidney (HEK293) Cells,” Biochemical and Biophysical Research Communications 446 (2014): 316–321. 24602615 10.1016/j.bbrc.2014.02.110

[36] P. Fang , B. He , M. Yu , et al., “Treatment With Celastrol Protects Against Obesity Through Suppression of Galanin‐Induced Fat Intake and Activation of PGC‐1alpha/GLUT4 Axis‐Mediated Glucose Consumption,” Biochimica et Biophysica Acta—Molecular Basis of Disease 1865 (2019): 1341–1350. 30742994 10.1016/j.bbadis.2019.02.002

[37] N. Canova , J. Sipkova , M. Arora , et al., “Effects of Celastrol on the Heart and Liver Galaninergic System Expression in a Mouse Model of Western‐Type Diet‐Induced Obesity and Metabolic Dysfunction‐Associated Steatotic Liver Disease and Steatohepatitis,” Frontiers in Pharmacology 16 (2025): 1476994. 39968178 10.3389/fphar.2025.1476994 PMC11832397

[38] M. Yu , M. Wang , S. Han , et al., “Spexin Ameliorates Skeletal Muscle Insulin Resistance Through Activation of GAL2 Receptor,” European Journal of Pharmacology 917 (2022): 174731. 34973950 10.1016/j.ejphar.2021.174731

[39] C. Y. Lin , L. Zhao , T. Huang , et al., “Spexin Acts as Novel Regulator for Bile Acid Synthesis,” Frontiers in Physiology 9 (2018): 378. 29692737 10.3389/fphys.2018.00378 PMC5902714

[40] K. Freimann , K. Kurrikoff , and U. Langel , “Galanin Receptors as a Potential Target for Neurological Disease,” Expert Opinion on Therapeutic Targets 19 (2015): 1665–1676. 26220265 10.1517/14728222.2015.1072513

[41] R. Lang , A. L. Gundlach , F. E. Holmes , et al., “Physiology, Signaling, and Pharmacology of Galanin Peptides and Receptors: Three Decades of Emerging Diversity,” Pharmacological Reviews 67 (2015): 118–175. 25428932 10.1124/pr.112.006536

[42] N. N. Rieser , M. Ronchetti , A. L. Hotz , and S. C. F. Neuhauss , “Guardian of Excitability: Multifaceted Role of Galanin in Whole Brain Excitability,” preprint, bioRxiv, 2024, 10.1101/2024.04.05.588285. PMC12227200 40613532

[43] C. Millon , A. Flores‐Burgess , M. Narvaez , et al., “The Neuropeptides Galanin and Galanin(1‐15) in Depression‐Like Behaviours,” Neuropeptides 64 (2017): 39–45. 28196617 10.1016/j.npep.2017.01.004

[44] X. Lu , A. Mazarati , P. Sanna , S. Shinmei , and T. Bartfai , “Distribution and Differential Regulation of Galanin Receptor Subtypes in Rat Brain: Effects of Seizure Activity,” Neuropeptides 39 (2005): 147–152. 15944003 10.1016/j.npep.2004.12.011

[45] J. G. Hohmann , A. Jureus , D. N. Teklemichael , A. M. Matsumoto , D. K. Clifton , and R. A. Steiner , “Distribution and Regulation of Galanin Receptor 1 Messenger RNA in the Forebrain of Wild Type and Galanin‐Transgenic Mice,” Neuroscience 117 (2003): 105–117. 12605897 10.1016/s0306-4522(02)00798-4

[46] M. Caramia , R. A. Romanov , S. Sideromenos , et al., “Neuronal Diversity of Neuropeptide Signaling, Including Galanin, in the Mouse Locus Coeruleus,” Proceedings of the National Academy of Sciences of the United States of America 120 (2023): e2222095120. 37487094 10.1073/pnas.2222095120 PMC10401028

[47] G. Juhasz , G. Hullam , N. Eszlari , et al., “Brain Galanin System Genes Interact With Life Stresses in Depression‐Related Phenotypes,” Proceedings of the National Academy of Sciences of the United States of America 111 (2014): E1666–E1673. 24706871 10.1073/pnas.1403649111 PMC4000859

[48] R. Lang , A. L. Gundlach , and B. Kofler , “The Galanin Peptide Family: Receptor Pharmacology, Pleiotropic Biological Actions, and Implications in Health and Disease,” Pharmacology & Therapeutics 115 (2007): 177–207. 17604107 10.1016/j.pharmthera.2007.05.009

[49] C. R. Elliott‐Hunt , R. J. Pope , P. Vanderplank , and D. Wynick , “Activation of the Galanin Receptor 2 (GalR2) Protects the Hippocampus From Neuronal Damage,” Journal of Neurochemistry 100 (2007): 780–789. 17263796 10.1111/j.1471-4159.2006.04239.x PMC2705497

[50] A. Tran , W. He , J. T. C. Chen , and D. D. Belsham , “Spexin: Its Role, Regulation, and Therapeutic Potential in the Hypothalamus,” Pharmacology & Therapeutics 233 (2022): 108033. 34763011 10.1016/j.pharmthera.2021.108033

[51] Y. Komuro , L. Galas , Y. M. Morozov , et al., “The Role of Galanin in Cerebellar Granule Cell Migration in the Early Postnatal Mouse During Normal Development and After Injury,” Journal of Neuroscience 41 (2021): 8725–8741. 34462307 10.1523/JNEUROSCI.0900-15.2021 PMC8528496

[52] E. G. Mills , C. Izzi‐Engbeaya , A. Abbara , A. N. Comninos , and W. S. Dhillo , “Functions of Galanin, Spexin and Kisspeptin in Metabolism, Mood and Behaviour,” Nature Reviews. Endocrinology 17 (2021): 97–113. 10.1038/s41574-020-00438-1 33273729

[53] P. Fang , Y. She , J. Zhao , et al., “Emerging Roles of Kisspeptin/Galanin in Age‐Related Metabolic Disease,” Mechanisms of Ageing and Development 199 (2021): 111571. 34517021 10.1016/j.mad.2021.111571

[54] Y. Kambe , T. T. Nguyen , T. Yasaka , et al., “The Pivotal Role of Neuropeptide Crosstalk From Ventromedial‐PACAP to Dorsomedial‐Galanin in the Appetite Regulation in the Mouse Hypothalamus,” Molecular Neurobiology 60 (2023): 171–182. 36251233 10.1007/s12035-022-03084-y

[55] A. Idelevich , K. Sato , K. Nagano , G. Rowe , F. Gori , and R. Baron , “Neuronal Hypothalamic Regulation of Body Metabolism and Bone Density Is Galanin Dependent,” Journal of Clinical Investigation 128 (2018): 2626–2641. 29596063 10.1172/JCI99350 PMC5983337

[56] M. Zalecki , W. Sienkiewicz , A. Franke‐Radowiecka , M. Klimczuk , and J. Kaleczyc , “The Influence of Gastric Antral Ulcerations on the Expression of Galanin and GalR1, GalR2, GalR3 Receptors in the Pylorus With Regard to Gastric Intrinsic Innervation of the Pyloric Sphincter,” PLoS One 11 (2016): e0155658. 27175780 10.1371/journal.pone.0155658 PMC4866767

[57] S. Gonkowski , P. Burlinski , C. Skobowiat , M. Majewski , and J. Calka , “Inflammation‐ and Axotomy‐Induced Changes in Galanin‐Like Immunoreactive (GAL‐LI) Nerve Structures in the Porcine Descending Colon,” Acta Veterinaria Hungarica 58 (2010): 91–103. 20159743 10.1556/AVet.58.2010.1.10

[58] K. Palus , K. Makowska , and J. Calka , “Alterations in Galanin‐Like Immunoreactivity in the Enteric Nervous System of the Porcine Stomach Following Acrylamide Supplementation,” International Journal of Molecular Sciences 20 (2019): 3345. 31288386 10.3390/ijms20133345 PMC6651480

[59] M. Bulc , K. Palus , L. Zielonka , M. Gajecka , and J. Calka , “Changes in Expression of Inhibitory Substances in the Intramural Neurons of the Stomach Following Streptozotocin‐Induced Diabetes in the Pig,” World Journal of Gastroenterology 23 (2017): 6088–6099. 28970724 10.3748/wjg.v23.i33.6088 PMC5597500

[60] K. Palus , M. Chmielewska‐Krzesinska , B. Jana , and J. Calka , “Glyphosate‐Induced Changes in the Expression of Galanin and GALR1, GALR2 and GALR3 Receptors in the Porcine Small Intestine Wall,” Scientific Reports 14 (2024): 8905. 38632282 10.1038/s41598-024-59581-8 PMC11024183

[61] E. Ekblad , A. Rokaeus , R. Hakanson , and F. Sundler , “Galanin Nerve Fibers in the Rat Gut: Distribution, Origin and Projections,” Neuroscience 16 (1985): 355–363. 2417157 10.1016/0306-4522(85)90008-9

[62] J. B. Furness , M. Costa , A. Rokaeus , T. J. McDonald , and B. Brooks , “Galanin‐Immunoreactive Neurons in the Guinea‐Pig Small Intestine: Their Projections and Relationships to Other Enteric Neurons,” Cell and Tissue Research 250 (1987): 607–615. 2446770 10.1007/BF00218954

[63] Y. F. Wang , Y. K. Mao , T. J. McDonald , and E. E. Daniel , “Distribution of Galanin‐Immunoreactive Nerves in the Canine Gastrointestinal Tract,” Peptides 16 (1995): 237–247. 7540291 10.1016/0196-9781(94)00170-7

[64] F. E. Bauer , A. Zintel , M. J. Kenny , D. Calder , M. A. Ghatei , and S. R. Bloom , “Inhibitory Effect of Galanin on Postprandial Gastrointestinal Motility and Gut Hormone Release in Humans,” Gastroenterology 97 (1989): 260–264. 2472997 10.1016/0016-5085(89)90059-0

[65] E. Feher and G. Burnstock , “Galanin‐Like Immunoreactive Nerve Elements in the Small Intestine of the Rat. An Electron Microscopic Immunocytochemical Study,” Neuroscience Letters 92 (1988): 137–142. 2460807 10.1016/0304-3940(88)90049-3

[66] T. Kiyohara , M. Okuno , H. Ishikawa , et al., “Galanin‐Induced Alteration of Electrolyte Transport in the Rat Intestine,” American Journal of Physiology 263 (1992): G502–G507. 1384358 10.1152/ajpgi.1992.263.4.G502

[67] D. D. Lorimer , K. Matkowskj , and R. V. Benya , “Cloning, Chromosomal Location, and Transcriptional Regulation of the Human Galanin‐1 Receptor Gene (GALN1R),” Biochemical and Biophysical Research Communications 241 (1997): 558–564. 9425310 10.1006/bbrc.1997.7838

[68] H. Nagase , A. Nakajima , H. Sekihara , D. A. York , and G. A. Bray , “Regulation of Feeding Behavior, Gastric Emptying, and Sympathetic Nerve Activity to Interscapular Brown Adipose Tissue by Galanin and Enterostatin: The Involvement of Vagal‐Central Nervous System Interactions,” Journal of Gastroenterology 37, no. S14 (2002): 118–127. 12572879 10.1007/BF03326430

[69] J. Fontaine and P. Lebrun , “Galanin: Ca2+‐Dependent Contractile Effects on the Isolated Mouse Distal Colon,” European Journal of Pharmacology 164 (1989): 583–586. 2475349 10.1016/0014-2999(89)90268-9

[70] S. Katsoulis , W. E. Schmidt , H. Schworer , and W. Creutzfeldt , “Effects of Galanin, Its Analogues and Fragments on Rat Isolated Fundus Strips,” British Journal of Pharmacology 101 (1990): 297–300. 1701674 10.1111/j.1476-5381.1990.tb12704.x PMC1917707

[71] I. Muramatsu and N. Yanaihara , “Contribution of Galanin to Non‐Cholinergic, Non‐Adrenergic Transmission in Rat Ileum,” British Journal of Pharmacology 94 (1988): 1241–1249. 2463026 10.1111/j.1476-5381.1988.tb11644.x PMC1854108

[72] K. Tamura , J. M. Palmer , and J. D. Wood , “Galanin Suppresses Nicotinic Synaptic Transmission in the Myenteric Plexus of Guinea‐Pig Small Intestine,” European Journal of Pharmacology 136 (1987): 445–446. 2440701 10.1016/0014-2999(87)90323-2

[73] K. A. Matkowskyj , R. Nathaniel , R. Prasad , D. Weihrauch , M. Rao , and R. V. Benya , “Galanin Contributes to the Excess Colonic Fluid Secretion Observed in Dextran Sulfate Sodium Murine Colitis,” Inflammatory Bowel Diseases 10 (2004): 408–416. 15475749 10.1097/00054725-200407000-00012

[74] T. Pham , S. Guerrini , H. Wong , J. Reeve, Jr. , and C. Sternini , “Distribution of Galanin Receptor 1 Immunoreactivity in the Rat Stomach and Small Intestine,” Journal of Comparative Neurology 450 (2002): 292–302. 12209857 10.1002/cne.10311

[75] S. Rattan and W. Tamura , “Role of Galanin in the Gastrointestinal Sphincters,” Annals of the New York Academy of Sciences 863 (1998): 143–155. 9928167 10.1111/j.1749-6632.1998.tb10691.x

[76] S. Siri , F. Maier , L. Chen , S. Santos , D. M. Pierce , and B. Feng , “Differential Biomechanical Properties of Mouse Distal Colon and Rectum Innervated by the Splanchnic and Pelvic Afferents,” American Journal of Physiology. Gastrointestinal and Liver Physiology 316 (2019): G473–G481. 30702901 10.1152/ajpgi.00324.2018 PMC6483024

[77] T. S. Taylor , P. Konda , S. S. John , D. C. Bulmer , J. R. F. Hockley , and E. S. J. Smith , “Galanin Suppresses Visceral Afferent Responses to Noxious Mechanical and Inflammatory Stimuli,” Physiological Reports 8 (2020): e14326. 31960596 10.14814/phy2.14326 PMC6971316

[78] S. M. Brunner , F. Reichmann , J. Leitner , et al., “Galanin Receptor 3 Attenuates Inflammation and Influences the Gut Microbiota in an Experimental Murine Colitis Model,” Scientific Reports 11 (2021): 564. 33436730 10.1038/s41598-020-79456-y PMC7803768

[79] D. Yoon , K. Bae , M. K. Lee , J. H. Kim , and K. A. Yoon , “Galanin Is an Epigenetically Silenced Tumor Suppressor Gene in Gastric Cancer Cells,” PLoS One 13 (2018): e0193275. 29462183 10.1371/journal.pone.0193275 PMC5819827

[80] A. D. Petrescu , S. Grant , E. Williams , et al., “Coordinated Targeting of Galanin Receptors on Cholangiocytes and Hepatic Stellate Cells Ameliorates Liver Fibrosis in Multidrug Resistance Protein 2 Knockout Mice,” American Journal of Pathology 190 (2020): 586–601. 31953035 10.1016/j.ajpath.2019.10.023 PMC7074378

[81] S. Huber , T. Fitzner , R. G. Feichtinger , et al., “Galanin System in the Human Bile Duct and Perihilar Cholangiocarcinoma,” Cells 12 (2023): 1678. 37443714 10.3390/cells12131678 PMC10340323

[82] M. Wang , Z. Zhu , Y. Kan , et al., “Treatment With Spexin Mitigates Diet‐Induced Hepatic Steatosis In Vivo and In Vitro Through Activation of Galanin Receptor 2,” Molecular and Cellular Endocrinology 552 (2022): 111688. 35654225 10.1016/j.mce.2022.111688

[83] N. Kuem , S. J. Song , R. Yu , J. W. Yun , and T. Park , “Oleuropein Attenuates Visceral Adiposity in High‐Fat Diet‐Induced Obese Mice Through the Modulation of WNT10b‐ and Galanin‐Mediated Signalings,” Molecular Nutrition & Food Research 58 (2014): 2166–2176. 25104077 10.1002/mnfr.201400159

[84] B. K. Smith , D. A. York , and G. A. Bray , “Chronic Cerebroventricular Galanin Does Not Induce Sustained Hyperphagia or Obesity,” Peptides 15 (1994): 1267–1272. 7531842 10.1016/0196-9781(94)90152-x

[85] S. F. Leibowitz , A. Akabayashi , and J. Wang , “Obesity on a High‐Fat Diet: Role of Hypothalamic Galanin in Neurons of the Anterior Paraventricular Nucleus Projecting to the Median Eminence,” Journal of Neuroscience 18 (1998): 2709–2719. 9502828 10.1523/JNEUROSCI.18-07-02709.1998 PMC6793124

[86] S. A. Jung , M. Choi , S. Kim , R. Yu , and T. Park , “Cinchonine Prevents High‐Fat‐Diet‐Induced Obesity Through Downregulation of Adipogenesis and Adipose Inflammation,” PPAR Research 2012 (2012): 541204. 22675336 10.1155/2012/541204 PMC3362995

[87] J. Wang , A. Akabayashi , H. J. Yu , et al., “Hypothalamic Galanin: Control by Signals of Fat Metabolism,” Brain Research 804 (1998): 7–20. 9729239 10.1016/s0006-8993(98)00632-5

[88] S. Cho , Y. Choi , S. Park , and T. Park , “Carvacrol Prevents Diet‐Induced Obesity by Modulating Gene Expressions Involved in Adipogenesis and Inflammation in Mice Fed With High‐Fat Diet,” Journal of Nutritional Biochemistry 23 (2012): 192–201. 21447440 10.1016/j.jnutbio.2010.11.016

[89] R. Y. Li , H. D. Song , W. J. Shi , et al., “Galanin Inhibits Leptin Expression and Secretion in Rat Adipose Tissue and 3T3‐L1 Adipocytes,” Journal of Molecular Endocrinology 33 (2004): 11–19. 15291739 10.1677/jme.0.0330011

[90] Y. Zhang , R. Proenca , M. Maffei , M. Barone , L. Leopold , and J. M. Friedman , “Positional Cloning of the Mouse Obese Gene and Its Human Homologue,” Nature 372 (1994): 425–432. 7984236 10.1038/372425a0

[91] A. Milewicz , B. Bidzinska , E. Mikulski , M. Demissie , and U. Tworowska , “Influence of Obesity and Menopausal Status on Serum Leptin, Cholecystokinin, Galanin and Neuropeptide Y Levels,” Gynecological Endocrinology 14 (2000): 196–203. 10923281 10.3109/09513590009167682

[92] S. M. Khan , O. P. Hamnvik , M. Brinkoetter , and C. S. Mantzoros , “Leptin as a Modulator of Neuroendocrine Function in Humans,” Yonsei Medical Journal 53 (2012): 671–679. 22665330 10.3349/ymj.2012.53.4.671 PMC3381496

[93] C. S. Yuan , L. Dey , J. T. Xie , and H. H. Aung , “Gastric Effects of Galanin and Its Interaction With Leptin on Brainstem Neuronal Activity,” Journal of Pharmacology and Experimental Therapeutics 301 (2002): 488–493. 11961047 10.1124/jpet.301.2.488

[94] Z. Shi , N. E. Pelletier , J. Wong , et al., “Leptin Increases Sympathetic Nerve Activity via Induction of Its Own Receptor in the Paraventricular Nucleus,” eLife 9 (2020): e55357. 32538782 10.7554/eLife.55357 PMC7316512

[95] A. L. Gundlach , “Galanin/GALP and Galanin Receptors: Role in Central Control of Feeding, Body Weight/Obesity and Reproduction?,” European Journal of Pharmacology 440 (2002): 255–268. 12007540 10.1016/s0014-2999(02)01433-4

[96] S. Hirako , N. Wada , H. Kageyama , et al., “Autonomic Nervous System‐Mediated Effects of Galanin‐Like Peptide on Lipid Metabolism in Liver and Adipose Tissue,” Scientific Reports 6 (2016): 21481. 26892462 10.1038/srep21481 PMC4759810

[97] N. J. Poritsanos , T. M. Mizuno , M. E. Lautatzis , and M. Vrontakis , “Chronic Increase of Circulating Galanin Levels Induces Obesity and Marked Alterations in Lipid Metabolism Similar to Metabolic Syndrome,” International Journal of Obesity 33 (2009): 1381–1389. 19773738 10.1038/ijo.2009.187

[98] C. Lawrence and G. S. Fraley , “Galanin‐Like Peptide (GALP) is a Hypothalamic Regulator of Energy Homeostasis and Reproduction,” Frontiers in Neuroendocrinology 32 (2011): 1–9. 20558195 10.1016/j.yfrne.2010.06.001 PMC2950899

[99] S. B. Sherman , M. Harberson , R. Rashleigh , et al., “Spexin Modulates Molecular Thermogenic Profile of Adipose Tissue and Thermoregulatory Behaviors in Female C57BL/6 Mice,” Hormones and Behavior 143 (2022): 105195. 35580373 10.1016/j.yhbeh.2022.105195 PMC10150790

[100] S. F. Morrison and C. J. Madden , “Central Nervous System Regulation of Brown Adipose Tissue,” Compr Physiol 4 (2014): 1677–1713. 25428857 10.1002/cphy.c140013 PMC4435534

[101] S. M. Schmidhuber , R. Santic , C. W. Tam , J. W. Bauer , B. Kofler , and S. D. Brain , “Galanin‐Like Peptides Exert Potent Vasoactive Functions In Vivo,” Journal of Investigative Dermatology 127 (2007): 716–721. 17024098 10.1038/sj.jid.5700569

[102] S. M. Schmidhuber , I. Rauch , B. Kofler , and S. D. Brain , “Evidence That the Modulatory Effect of Galanin on Inflammatory Edema Formation Is Mediated by the Galanin Receptor 3 in the Murine Microvasculature,” Journal of Molecular Neuroscience 37 (2009): 177–181. 18679831 10.1007/s12031-008-9135-x

[103] J. W. Bauer , R. Lang , M. Jakab , and B. Kofler , “Galanin Family of Peptides in Skin Function,” Cellular and Molecular Life Sciences 65 (2008): 1820–1825. 18500644 10.1007/s00018-008-8156-5 PMC11131780

[104] Y. Liu , L. Sun , L. Zheng , et al., “Spexin Protects Cardiomyocytes From Hypoxia‐Induced Metabolic and Mitochondrial Dysfunction,” Naunyn‐Schmiedeberg's Archives of Pharmacology 393 (2020): 25–33. 31396649 10.1007/s00210-019-01708-0

[105] W. Ou , H. Liu , C. Chen , et al., “Spexin Inhibits Excessive Autophagy‐Induced Ferroptosis to Alleviate Doxorubicin‐Induced Cardiotoxicity by Upregulating Beclin 1,” British Journal of Pharmacology 181 (2024): 4195–4213. 38961632 10.1111/bph.16484

[106] D. Li , Y. Liu , C. Li , et al., “Spexin Diminishes Atrial Fibrillation Vulnerability by Acting on Galanin Receptor 2,” Circulation 150 (2024): 111–127. 38726666 10.1161/CIRCULATIONAHA.123.067517

[107] B. Jana , J. Calka , and B. Micinski , “Regulatory Influence of Galanin and GALR1/GALR2 Receptors on Inflamed Uterus Contractility in Pigs,” International Journal of Molecular Sciences 22 (2021): 6415. 34203944 10.3390/ijms22126415 PMC8232690

[108] F. Azin and H. Khazali , “Neuropeptide Galanin and Its Effects on Metabolic and Reproductive Disturbances in Female Rats With Estradiol Valerate (EV)—Induced Polycystic Ovary Syndrome (PCOS),” Neuropeptides 80 (2020): 102026. 32063381 10.1016/j.npep.2020.102026

[109] B. S. Kilic , N. Atakul , S. Selek , and Y. Atamer , “Maternal Neuropeptide Galanin Levels in Pregnancies With Intra‐Uterine Growth Restriction (IUGR): Neurohormonal Regulation of Fetal Weight,” Irish Journal of Medical Science 192 (2023): 1259–1264. 35996067 10.1007/s11845-022-03132-5

[110] P. Yuan , X. Li , W. J. Xiong , J. Jiang , and R. Jiang , “Downregulation of the Expression of Galanin Impairs Erectile Function in Hypoandrogenic Rats,” Sexual Medicine 11 (2023): qfad029. 37351545 10.1093/sexmed/qfad029 PMC10281959

[111] I. Rauch , L. Lundstrom , M. Hell , W. Sperl , and B. Kofler , “Galanin Message‐Associated Peptide Suppresses Growth and the Budded‐to‐Hyphal‐Form Transition of *Candida albicans* ,” Antimicrobial Agents and Chemotherapy 51 (2007): 4167–4170. 17698619 10.1128/AAC.00166-07 PMC2151459

[112] O. Cordero‐Llana , F. Rinaldi , P. A. Brennan , D. Wynick , and M. A. Caldwell , “Galanin Promotes Neuronal Differentiation From Neural Progenitor Cells In Vitro and Contributes to the Generation of New Olfactory Neurons in the Adult Mouse Brain,” Experimental Neurology 256 (2014): 93–104. 24726665 10.1016/j.expneurol.2014.04.001

[113] A. M. El‐Bareg , Regulation of Galanin in Human Embryos and Human Embryonic Stem Cells (University of Manchester, 2007), p. 290.

[114] Z. Zhang , P. Fang , M. Shi , et al., “Association Between Circulating Levels of Galanin and Pre‐Pregnancy Body Mass Index in Patients With Gestational Diabetes Mellitus,” Eating Behaviors 19 (2015): 57–60. 26172564 10.1016/j.eatbeh.2015.06.003

[115] B. Kleine , S. Wolfahrt , M. Lotsch , T. Gantner , and W. G. Rossmanith , “Expression of Galanin in Human Placenta,” Molecular Human Reproduction 7 (2001): 379–385. 11279301 10.1093/molehr/7.4.379

[116] J. Zamlynski , J. Chudek , A. Olejek , et al., “Galanin Concentrations in Maternal Circulation, Amniotic Fluid and Umbilical Cord Blood During Term Labor: Relationship With Maternal Body Mass and Neonatal Birth Weight,” Gynecological Endocrinology 23 (2007): 295–299. 17558689 10.1080/09513590701281405

[117] S. Falkenstetter , J. Leitner , S. M. Brunner , T. N. Rieder , B. Kofler , and S. Weis , “Galanin System in Human Glioma and Pituitary Adenoma,” Frontiers in Endocrinology 11 (2020): 155. 32265844 10.3389/fendo.2020.00155 PMC7105811

[118] M. L. Sanchez and R. Covenas , “The Galaninergic System: A Target for Cancer Treatment,” Cancers (Basel) 14 (2022): 3755. 35954419 10.3390/cancers14153755 PMC9367524

[119] S. Huber , T. Fitzner , R. G. Feichtinger , et al., “Spexin Expression in the Human Bile Duct and Perihilar Cholangiocarcinoma,” Peptides 188 (2025): 171405. 40194702 10.1016/j.peptides.2025.171405

[120] L. Stevenson , W. L. Allen , R. Turkington , et al., “Identification of Galanin and Its Receptor GalR1 as Novel Determinants of Resistance to Chemotherapy and Potential Biomarkers in Colorectal cancer,” Clinical Cancer Research 18 (2012): 5412–5426. 22859720 10.1158/1078-0432.CCR-12-1780 PMC3463501

